# Internet-delivered cognitive behavioral therapy (iCBT) for common mental disorders and subsequent sickness absence: a systematic review and meta-analysis

**DOI:** 10.1177/14034948221075016

**Published:** 2022-02-04

**Authors:** Lina Udd-granat, Jouni Lahti, Michael Donnelly, Charlene Treanor, Sami P. Pirkola, Tea Lallukka, Anne Kouvonen

**Affiliations:** 1 Department of Public Health, Faculty of Medicine, University of Helsinki, Finland; 2 Faculty of Social Sciences, University of Helsinki, Finland; 3 Centre for Public Health, Queen’s University Belfast, Northern Ireland; 4 Faculty of Social Sciences, Tampere University, Finland; 5 Department of Psychiatry, Tampere University Central Hospital, Finland

**Keywords:** Mental health, sick leave, absenteeism, cognitive behavioral therapy, telemedicine

## Abstract

**Aim:**

The study aimed to critically review and synthesize the best available evidence about the effectiveness of therapist-guided internet-delivered cognitive behavioral therapy (iCBT) in terms of reducing sickness absence (SA).

**Methods:**

We searched Medline (PubMed), Embase, PsycInfo, CINAHL, and Cochrane Central (up to November 2020) for English language peer-reviewed papers that described randomized controlled trials of therapist-guided iCBT compared with usual treatment for SA in adults with common mental disorders. Eligible studies were assessed with the Cochrane Risk of Bias 1 tool, meta-analysis was conducted using a random-effects model, and standardized mean differences (SMD) with 95% confidence intervals (CI) were reported. A subgroup analysis investigated potential moderating variables (diagnosis, SA at baseline, and estimated accuracy of self-report).

**Results:**

We identified 2788 references, of which 68 remained after the completion of the systematic screening process. A hand search of reference lists yielded no additional studies. The full texts of these 68 studies were appraised critically, and 11 were deemed to be suitable for a meta-analysis. SA was similar for iCBT and usual treatment groups (SMD: 0.02, 95% CI, –0.08 to 0.11), and remained similar even after the removal of two studies in which the recall time was over 3 months (SMD: 0.00, –0.11 to 0.12). Similar SA levels in intervention and control groups at 6-month and 12-month follow-up were observed in studies of participants with depression symptoms.

**Conclusions:**

**iCBT did not appear to be effective in terms of reducing (largely self-assessed) SA in adults with common mental disorders. There is a need to improve the method and consistency of assessing SA.**

## Introduction

Common mental disorders (CMDs) are significant contributors increasingly to the global burden of disease [1]. Conditions such as insomnia, anxiety, and depression among the working-age population are the subject of concentrated public health efforts because they impact negatively on performance at work, including leading to an inability to work as well as affecting the quality of life of people with CMDs [2,3]. Sickness absence (SA) may be viewed in terms of indicating a potential means of harm reduction from the perspective of an employer and an employee given the risk of accidents and disruptions in workplaces.

Furthermore, SA has direct negative economic consequences for workers with mental ill-health, employers, and society at large [4], and indirect costs through losses regarding the wellbeing of colleagues [5]. Staying at work when the working capacity of a worker is reduced is becoming socially acceptable – a behavioral trend that may cause over-reporting and an overestimation of the negative impact of this presenteeism in comparison with SA on work flow and productivity [6]. Working in stressful environments can lead to the onset, or promote the progression, of common mental disorders [7], while on the other hand, protective factors in the workplace such as positive support can contribute to better physical and mental health functioning [8]. Returning to work or employment has been associated with better long-term mental well-being [9]. Workplace interventions that were aimed directly at facilitating people with depression to return to work had positive short-term effects but they did not significantly reduce SA in the long run [10]. Face-to-face or Internet-delivered psychological interventions may reduce the number of SA days, although there is a need to test this claim with robust studies [10]. In addition, physical exercise has shown some promise in this regard [10]. Successful return to work after sick-leave due to mental illness is affected by a wide array of factors (as well as disease severity) ranging from individual characteristics to workplace environment [11]. The effective and timely treatment of the mental ill-health condition underlying a SA is recognized as an important route to supporting an individual to stay employed and working [12].

The search for readily available and cost-efficient treatment approaches to the treatment of common mental disorders led to the development of Internet-delivered therapies, especially Internet-delivered cognitive behavioral therapy (iCBT). Several systematic reviews point to a body of evidence indicating the effectiveness of iCBT at least in terms of symptom relief [13–15]. Guided online therapy has been deemed to be safer than treatment as usual regarding the risk of symptom deterioration during treatment [16]. Internet delivered CBT has been identified as an effective treatment for symptoms of depression and anxiety [13,17,18], insomnia [19], and stress-related symptoms [20], although the economic impacts of iCBT have not been appraised or systematically reviewed. A simulation or modelling of economic costs in 2020 [18] pointed to the cost-efficiency of iCBT over traditional face-to-face CBT, due mainly to reduced waiting times and, thus, less time in an untreated disease state. However, cost-efficiency studies have tended to focus on the direct costs of therapists, medication, and hospital admission without considering SA and presenteeism as major cost drivers [21].

This study aimed to critically review and synthesize the best available evidence about the effectiveness of therapist-guided iCBT in terms of reducing SA. This is the first systematic review and meta-analysis of studies of iCBT interventions that focused on common mental disorders, reported SA, and examined whether iCBT offered any advantages or disadvantages in terms of lost workdays.

## Methods

The PRISMA (Preferred Reporting Items for Systematic Reviews and Meta-Analyses) standards were followed throughout the process (please see the supplemental PRISMA checklist) and the study protocol was registered with PROSPERO (CRD42020151604) before proceeding with the systematic searches.

### Eligibility criteria

Trials were eligible for inclusion in the review if they included an adult (18–64 years old) sample with clinically determined common mental health conditions or sleep disorders, particularly insomnia (WHO International Classification of Disease, version 10 codes F40–F42 for neurotic disorders, F32–39 for depressive disorders, F43.2 for adjustment disorders and F51.0 for insomnia). These codes cover phobic anxiety disorders, other anxiety disorders, obsessive-compulsive disorder, depressive episode, recurrent depressive disorder, persistent mood disorders, other mood disorders, and unspecified mood disorders. In addition, we included studies of adults with self-reported mental illness if they met the definition of a “case” as defined according to an empirically verified cut-off point on a validated scale. Trials were excluded if patients had psychiatric disorders outside this pre-defined group of diagnoses, for example, personality disorder, substance abuse, severe post-traumatic stress disorder, psychotic disorders, learning disability, attention deficit, or other neurodevelopmental/neuropsychiatric disorders. Also, studies that focused solely on seniors, children, or adolescents were excluded.

We excluded patients with bipolar and axis I disorders, post-traumatic stress disorder, neuropsychiatric disorders, and neurologic comorbidities disorders and substance abuse due to general differences in treatment and prognosis. Furthermore, there is some evidence to suggest that guided internet-based interventions may be superior to unguided interventions [22], thus we limited this review to therapist-guided interventions.

Outcome was SA for any cause, as mental illnesses such as depression and anxiety increase the risk of all types of health-related absence [2,3]. Uncertainty about the number and quality of studies that addressed SA and iCBT led to a decision to include in the review protocol (https://www.crd.york.ac.uk/prospero/display_record.php?RecordID=151604) a range of comparators (treatment as usual/waitlist/information control or placebo) and face-to-face CBT as well as intervention evaluations with various degrees of control in their study designs. The volume of studies was much larger than expected and the protocol was revised (purely for review project management reasons) to cover only randomized controlled trials (RCTs) of iCBT versus treatment as usual/waitlist/information control or placebo.

An RCT was eligible if the intervention consisted of therapist guided, internet-delivered (cognitive behavioral) therapy, in an outpatient, primary care, or similar, setting, in which patients were exposed to the same intervention components that comprise conventional CBT, but the material, instructions and assistance were provided through an Internet-based treatment platform. Assistance by telephone did not lead to the exclusion of a study, and studies with or without combined [selective serotonin reuptake inhibitors/serotonin and norepinephrine reuptake inhibitors (SSRI/SNRI)] medication and a variable number of sessions were eligible. The comparator was a wait-list or care-as-usual control group or a face-to-face CBT intervention group of working age adults.

In addition, studies were required to report data on post-intervention self-reported and/or confirmed SA (or similar, health-related absenteeism) to be eligible for inclusion in the review. We included studies that measured other outcomes such as work ability, work satisfaction, work functioning, and unemployment as long as they measured SA outcome. Only English language papers in the specified databases were included in the review.

### Information sources and search strategy

Medline through PubMed (1966–present), Embase (1966–present), PsycINFO (1967–present), CINAHL (1937–present), and Cochrane Central Register of Controlled Trials (1992–present), were searched in November 2020. Please see online supplemental material (PRISMA checklist) for a full description of the search terms and strategies that were applied to the databases.

Results of the database searches were imported into the Covidence bibliographic format [23]. Two authors screened independently titles and abstracts of all papers returned from the searches of databases. Conflicting views about the eligibility of papers (105 out of 114) were resolved by internal discussion or via discussion with a third author.

Ten full texts were obtained through direct contact with the authors (please see PRISMA flowchart and checklist as supplemental files). Two members of this review team reviewed the full texts independently against the inclusion and exclusion criteria, and disagreements were resolved by a third author. Papers based on the same trial were merged. The review team’s psychiatrist studied the reported descriptions of interventions and excluded two studies because it was unclear whether or not they were CBT-based [24,25]. Two studies were omitted due to incomplete outcome data [26], or study data was based on combined guided and unguided iCBT intervention [27].

### Data extraction

Data was extracted according to the Cochrane handbook for systematic reviews of interventions (Version 6.0.Wiley, 10/2019; www.cochrane-handbook.org).

Two review authors created an initial data extraction form or template using Covidence. The template was finalized after the review team discussed the data categories, the data that needed to be extracted, and the results from piloting its use with one of the included studies. Numerical data on SA was extracted in as much detail as possible and authors of the studies were contacted for original aggregate data. It was not possible to retrieve such data for a few studies – one study was omitted after communication with the study authors [26]; in another study [28], the standard deviation of the cost of absenteeism and presenteeism were combined and presented as an estimate for the standard deviation of absenteeism alone, and a calculated coefficient (21.5EUR/day) was used to calculate the baseline number of SA days; in a third study [29], the missing group mean and standard deviation for a time point of 3 months after baseline were reconstructed based on estimated values for SA categories that were presented in the appendices of a related article about the same study [30]; none = 0, low = 1.5, some = 5, high = 19, long-term = 30 days/month. This reconstructed dataset generated substantial heterogeneity in the meta-analysis and it was therefore omitted from the final analysis.

### Assessment of bias

**Figure 1.**
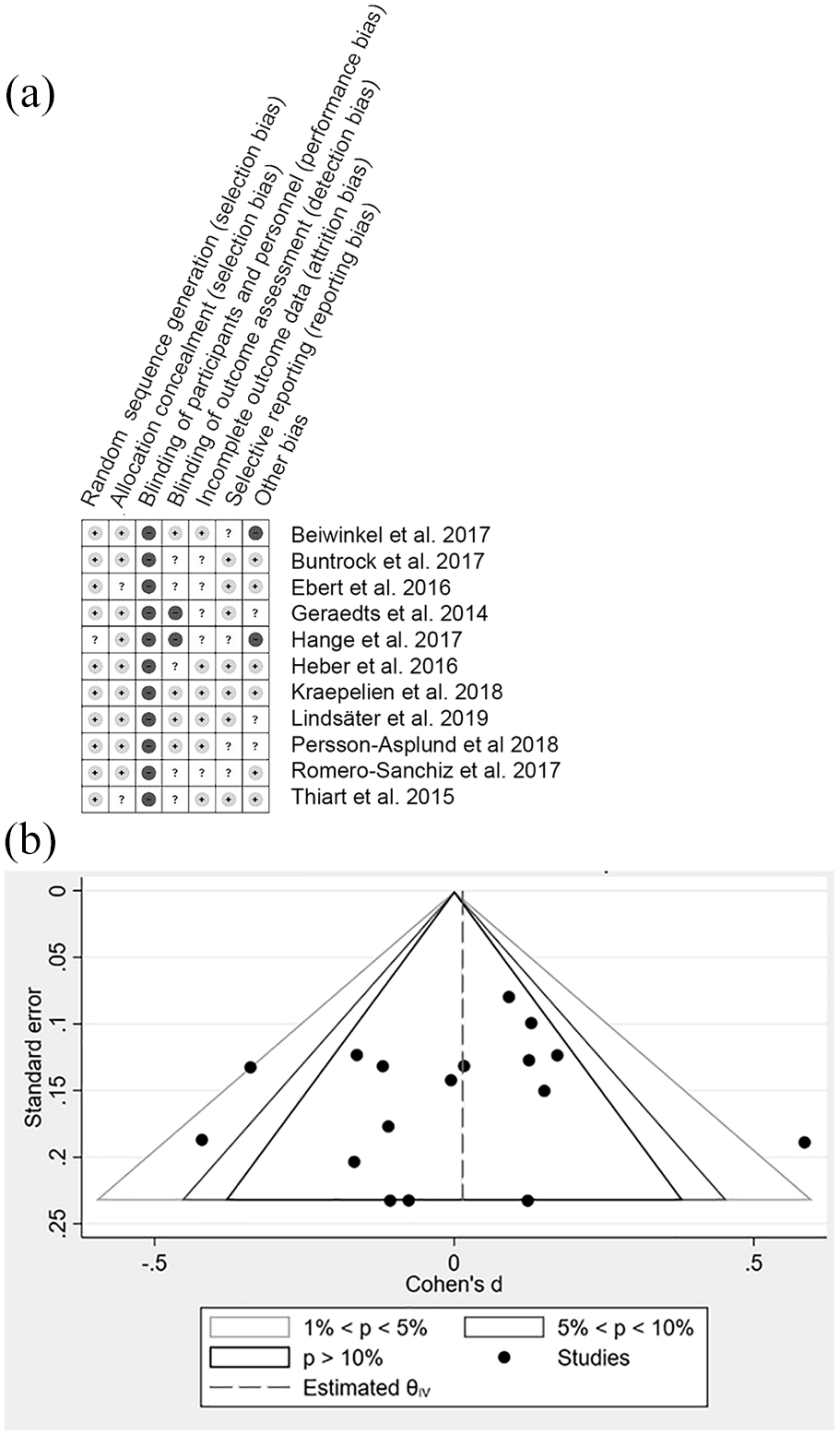
(a) Risk of bias assessment of the included studies. Light grey (+): low risk, white (?): unsure risk, dark grey (–): high risk. None of the studies attempted blinding of the participants (third column), due to the nature of the intervention, this is not necessarily good a marker of the study quality. Here, we suggest emphasis on the outcome assessment and possible detection bias (fourth column) due to self-reporting, recall times, and the social pressure during data collection (as written questionnaire (with or without predefined answering options) or during interview). (b) Funnel plot of all the included study timepoints, slight or no asymmetry.

The Cochrane Risk of Bias tool included in the Covidence format [31] was used independently by two authors to assess the quality of each included study and any bias in the assessment of the SA outcome. Conflicting views were resolved via discussion with a third review author. The risk of bias assessment in the form of “traffic lights” is displayed in Figure 1a and a detailed appraisal of the assessment of the SA outcome for accuracy and completeness is presented in Table I. Publication bias was assessed with funnel plots (Figure 1b). Visual inspection and an Egger’s test were used to evaluate the symmetry of the funnel plot and the trim-and-fill method was used to correct for possible publication bias in Stata (StataCorp 2019; Stata Statistical Software: Release 16, College Station, TX).

### Meta-analysis

Meta-analysis was performed when PICO elements were comparable and related data was available, in the RevMan5 software [32]. We used a random effects model to analyze data, and we used Cohen’s d or standardized mean differences (SMD) and 95% confidence intervals (CIs) regarding treatment effect sizes [10].

### Analysis of moderating effects of diagnosis and baseline SA

Baseline SA is a potential source of bias as SA is a characteristic of the population studied and a post-intervention outcome; thus, SA at one time point may affect the risk of SA at another time point [33]. Moreover, normative levels of SA may vary across different populations and countries [34]. This variation, which may be due to differences in the social and economic framework supporting SA, should be taken into account when extrapolating results from studies performed in one country to another country.

Firstly, we examined whether the SA at baseline in a study was above or below the relevant national average (Table II), by dividing the baseline mean number of SA days with the recall time for collecting the outcome and then, in turn, dividing this result with the respective national average of SA days published by the Organization for Economic Co-operation and Development (OECD) [34]. In this way, the population average was set to a value of 1.0 days per month. A meta-analysis was applied to studies that indicated “high SA” above 1.0 days per month, and “low SA” below this limit. All SA data from the studies are presented in the form of these standardized monthly mean days of SA in Table I for further comparability. The data are informed by communication with Buntrock et al. [36], Lindsäter et al. [20], Hange et al. [38], and Kraepelien et al. [29] (for baseline and 12-months) and calculated from SA costs for Romero-Sanchiz et al. [28], (SA costs divided with the value 21.5 EUR/SA day mentioned in the article as basis for the cost estimates). The post-intervention time point of Kraepelien et al. [29] is based on the mean number of days/month of SA in a given category (categories were denoted as follows: none = 0 days/month, low = 1–2 days/month, mean 1.5 days/month, some = 3–7 days/month, mean 5 days/month high = 8–30 days/month, mean 19 days/month, long term = 30 days/month) multiplied by the number of participants in a category, and the sum of such calculated days from all categories divided by the total number of study group participants [30]. Other standardized monthly means were calculated as described above using the data on SA days published in the respective articles.

## Results

### Characteristics of the included studies

All 11 included studies were conducted in Europe within the last decade (Supplemental Table S1, characteristics of studies), and 10 out of 11 were secondary analyses of trials that were designed to address intervention effectiveness. Only Beiwinkel et al. [35] reported SA as the primary aim or outcome. Power calculations were not performed in any study to evaluate the outcome of SA. Six studies focused on depression [28,29,35–38], and the rest assessed different stress-related mental health problems [16,20,39,40], or insomnia [19]. The majority (63%–85.6%) of participants were female and, in all but three studies [28,29,38], at least half had a university education. Six studies included only employed participants [16,19,37–40], while the other studies also included participants who were studying or absent from work for other reasons. SA data were collected primarily through the Trimbos/iMTA questionnaire for costs associated with psychiatric illness, “Treatment Inventory of Costs in Patients with psychiatric disorders (TiC-P),” although also other questionnaires were used for self-report [28,38]. Only one study obtained registry data about SA days [35]. The baseline level of SA among participants varied as some interventions were preventive in aims and orientation [16,36,37,39], whilst other interventions were described clearly as treatments. Four studies reported conflicting financial interests in the study intervention [16,19,36,39].

### The overall effect of iCBT on SA

Heterogeneity analysis of the full set of data from all time points in the studies revealed an I^2^ value of 50%, suggesting moderate heterogeneity. The funnel plot for all study time points (17 data sets) was skewed by one study and suggested that there may be some publication bias towards favoring publication of reduced SA in the intervention group or condition (Figure 1b). Egger’s t-test also indicated possible publication bias and no evidence of small study effects (*P* = 0.60). An indicative meta-analysis of SA data from all time points in every study showed a statistically non-significant SMD of 0.0 (95% CI –0.1 to 0.1) (supplemental Figure S1) and an analysis of the longest follow-up time point in each study (I^2^ of 26%) resulted in a non-significant SMD of 0.02 (95% CI –0.08 to 0.11). iCBT did not appear to have a positive or negative effect on SA compared with treatment as usual for people with the common mental disorders represented in the studies. A similar statistically non-significant total SMD of 0.00 (–0.11 to 0.12) was found when standardized mean SAs of study arms were compared (in the nine studies that were deemed to have recorded SA with at least moderate accuracy as described in Table I).

**Table I.** Quality assessment of the SA outcome.

Study ID [ref]	SA outcome quality	Supporting notes
Beiwinkel et al. 2017 [35]	High	Register SA data, low risk of reporting inaccuracy, data available for 89% of participants (despite otherwise very high attrition 69% (I), 66%(C))
Buntrock et al. 2017 [36]	Moderate	Self-reported SA data, moderate risk of reporting inaccuracy (3-month recall time), attrition intermediate (20–30%)
Ebert et al. 2016 [16]	Moderate	Self-reported SA data, moderate risk of reporting inaccuracy (3-month recall time), attrition intermediate (ca. 25%)
Heber et al. 2016 [39]	Moderate	Self-reported SA data, moderate risk of reporting inaccuracy (3-month recall time), attrition low (10%)
Thiart et al. 2016 [19]	Moderate	Self-reported SA data, moderate risk of reporting inaccuracy (3-month recall time), attrition low (8% (I), 14%(C))
Kraepelien et al. 2018 [29]	Moderate	Self-reported SA data, low risk of reporting inaccuracy (1-month recall time) for 12-month timepoint, intermediate risk for 3-month time point (in Kaldo et al. 2018), attrition high (38% (I), 44% (C))
Hange et al. 2017 [38]	Low	Self-reported SA data, high risk of reporting inaccuracy (up to 12-month recall time), attrition intermediate (ca. 25% (I), 20% (C)), reporting ambiguities
Lindsäter et al. 2019 [20]	High	Self-reported SA data, low risk of reporting inaccuracy (1-month recall time), attrition low (3% of all participants)
Persson Asplund et al. 2018 [40]	High	Self-reported SA data, low risk of reporting inaccuracy (1-month recall time), attrition intermediate (ca. 30%)
Geraedts et al. 2014 [37]	Low	Self-reported SA data, high risk of reporting inaccuracy (up to 6-month recall time), attrition intermediate at posttreatment (26%) and 6 months (32%), high (46%) at 12 months
Romero-Sanchiz et al. 2017 [28]	Moderate	Self-reported SA data, intermediate risk of reporting inaccuracy (3-month recall time), attrition intermediate ca. 30%

(C), control; (I), iCBT; iCBT, internet-delivered cognitive behavioral therapy; SA, sickness absence.

### Long-term time points

**Figure 2.**
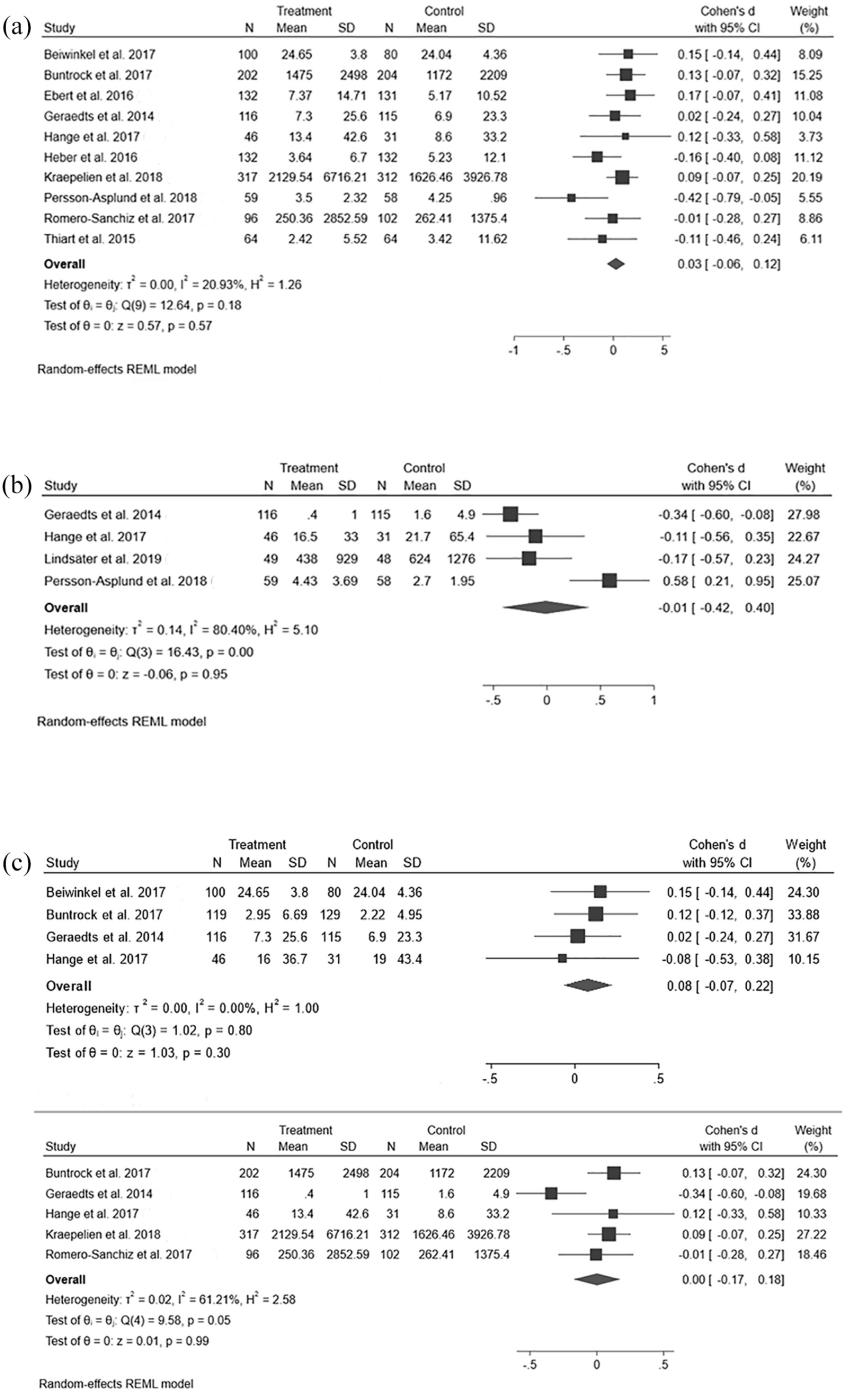
(a) iCBT effect on SA after intervention (during follow-up). (b) iCBT effect on SA during intervention. (c) iCBT effect on SA in studies of depression. Studies in alphabetical order, with all 6-month timepoints presented as separate lines of data above and all 12-month timepoints below.

SA outcome data from follow-up time points, that is, SA after the intervention program had been completed, were available for 10 out of 11 studies (for 6–12 months or both after baseline, depending on the study). The longest follow up time points of each study were comparable with a low heterogeneity, (I^2^ = 21%), and revealed a total SMD of 0.03 (95% CI –0.06 to 0.12), see Figure 2a.

### Post-intervention time points

Analysis of the post-intervention time points 2–3 months from baseline (SA during the intervention, see Figure 2b) identified three studies that might be interpreted as providing tentative support for the intervention [20,37,38] and one study that appeared to support the control condition [40]. The studies were statistically heterogeneous, with an I^2^ of 82% (no overlap between the confidence intervals of the effect sizes), and technically heterogeneous, as the study in which the result favored the control group differed from the other studies in terms of offering an “attention control” condition that comprised intense follow-up and online peer-group activities had a total SMD for the control group that was still close to zero (total SMD –0.01, 95% CI –0.42 to 0.40).

### Depression

A meta-analysis of the six studies that comprised participants with depression had low heterogeneity (I^2^ of 20%). The total SMD was statistically non-significant (SMD: 0.02, 95% CI -0.07—0.11). Similar results were observed when these data were analyzed according to the 6-month follow-up time point (Figure 2c) with a total SMD of 0.03 (95% CI –0.11 to 0.18) and 12-month follow-up (SMD: 0.08, –0.02 to 0.18, Figure 2c). A possible reduction in post-intervention SA was observed with a total SMD of –0.28 (–0.51 to 0.06) but an effect was not evident when post-intervention data from studies other than depression were added [20,40] as mentioned above, and also when the reconstructed post-intervention data from the study by Kraepelien et al. [29] (see Table II or Supplemental Figure S1) was added to the analysis.

**Table II.** Normalized monthly SA.

Study	Baseline	Post-intervention	6-month follow-up	12-month follow-up
(I) Beiwinkel et al. 2017 [35]	5.11	-	4.92	-
(C) Beiwinkel et al. 2017 [35]	5.51	-	4.80	-
(I) Romero-Sanchiz et al. 2017 [28]	4.98	-	-	3.76^b^
(C) Romero-Sanchiz et al. 2017 [28]	5.70	-	-	3.95^b^
(I) Kraepelien et al. 2018 [29]	3.54	2.67	-	3.92
(C) Kraepelien et al. 2018 [29]	3.28	1.63	-	3.54
(I) Lindsäter et al. 2019 [20]	3.41	3.84	4.73	-
(C) Lindsäter et al. 2019 [20]	4.07	3.59	-	-
(I) Hange et al. 2017 [38]	2.76	5.85	5.67	2.37
(C) Hange et al. 2017 [38]	1.10	7.69	6.73	1.52
(I) Persson Asplund et al. 2018 [40]	2.13	4.71^a^	3.72	-
(C) Persson Asplund et al. 2018 [40]	4.05	2.87^a^	4.52	-
(I) Thiart et al. 2016 [19]	1.11	-	0.49	-
(C) Thiart et al. 2016 [19]	1.04	-	0.68	-
(I) Heber et al. 2016 [39]	0.98	-	0.73	-
(C) Heber et al. 2016 [39]	0.87	-	1.04	-
(I) Ebert et al. 2016 [16]	0.97	-	1.47	1.17
(C) Ebert et al. 2016 [16]	0.72	-	1.03	-
(I) Geraedts et al. 2014 [37]	0.65	0.22^a^	0.98	1.33
(C) Geraedts et al. 2014 [37]	0.73	0.87^a^	1.36	1.25
(I) Buntrock et al. 2017 [36]	0.65	-	0.59	0.84
(C) Buntrock et al. 2017 [36]	0.55	-	0.44	0.64

The table figures are the mean number of SA days per month in the treatment groups (I) and (C) at the described timepoints divided by the national monthly average of SA days. The national monthly averages were retrieved from the OECD website for the year 2018, monthly average Germany 1.67, Sweden 0.94, Netherlands 0.92, Spain 1.03.

a 2 months, all others 3 months

b Based on intention-to-treat analysis (completers analysis 5.24(I), and 4.53 (C)).(C), control; (I), iCBT; iCBT, internet-delivered cognitive behavioral therapy; OECD, Organization for Economic Co-operation and Development; SA, sickness absence.

### Baseline SA

Control of the baseline SA indicators did not affect SA scores after receipt of an iCBT intervention. The studies with baseline SA above the national average had a total SMD of –0.02 (95% CI –0.15 to 0.12), I^2^ = 30% and studies below the national average had a total SMD of 0.05 (95% CI –0.10 to 0.19), I^2^ = 35% (see the forest plots in supplemental Figure S2).

## Discussion

The main finding of this study is that SA among patients with common mental disorders, mainly depression, does not appear to be reduced significantly by iCBT. This is the first systematic review to address SA as a key outcome of interest in the evaluation of iCBT for people with common mental disorders. There is a need for RCTs that are designed and powered to test the effectiveness of iCBT (and other mental ill-health treatments) in terms of SA as a primary endpoint or outcome.

A previous review investigated the effectiveness of psychological treatments and return-to-work interventions for common mental disorders [12], including some studies of iCBT interventions [16,19,37], but did not undertake or present a separate analysis for each treatment modality. In 2020, a review evaluated the effectiveness of work-based interventions, psychological interventions and medications for depression [10]. This latter review included guided iCBT as a subgroup but captured only two of the studies that were reviewed here [35,38]. It also included three studies that we decided to omit. The first of these was excluded because it did not distinguish between patients who received guided (~40%) and unguided (~60%) iCBT [27], the second because it delivered real-time online sessions with a therapist rather than multimedia modules [41], and the third because it did not contain the features that mark out a iCBT intervention, i.e., it was a computer program that patients accessed at a treatment center rather than being an Internet-delivered intervention [42]. The reviews concluded that CBT/iCBT conveyed a slight positive effect in terms of reducing SA. Arguably, the null finding of this review is based on the analysis of a more clearly delineated data set. It is likely that the discordant conclusions between the reviews are due, at least partly, to differences in eligibility criteria regarding the definition, and type of iCBT intervention. Future individual studies and reviews should aim to use standardized reporting guidelines for the description and evaluation of psychological interventions and treatments (e.g., [43]).

The outcome of this review should be interpreted mindful of several limitations. SA is not a primary endpoint or a commonly assessed outcome in clinical trials of iCBT judging from the relatively small number of studies that were included in this review compared with other systematic reviews of studies about the effectiveness of iCBT [13,15]. None of the trials in this review were designed primarily to address SA. Sometimes SA is reported independently, as a measure of symptom severity [37,38], but often it is used only as a form of contributing material that aids the calculation of costs and the determination of an index of cost-efficiency/cost-utility [20,28,29]. Power calculations were not undertaken to detect differences in SA. In one study, a power or sample size was calculated post hoc [20] and it indicated that the trial did not have enough power to detect between group/condition differences in SA. A strength of meta-analysis is that it has the potential to increase statistical power [44]. Regarding this meta-analysis, a retrospective power calculation indicates that based on 11 studies and a mean number of 106 participants per study group, a summary effect size larger than 0.4 would be detectable at approximately 99% power or higher [45]. The smallest summary effect size that would be detectable with an acceptable level of power (80%) would be 0.1645. Although this review contained a relatively small number of original studies, it included enough studies (and study participants), arguably, to overcome any sample size deficits in the original studies. The lack of attention that most studies gave to SA as a primary outcome, and that this fact meant that sample size was not calculated to provide adequate power to test this endpoint, may also be considered an important finding by itself and points to a need for further research guidelines.

Secondly, it is reasonable to argue that, in the current context, a register-based SA outcome may be regarded as a gold standard. Registry data, however, may be unavailable for ethical or practical reasons, and only absences above or below certain day limits may be recognized. This review found that most studies (10 out of11) used self-reports to determine SA. Self-reporting of SA may be subject to bias and under-reporting, and accuracy tends to decrease as recall duration time increases [6]. Aggregated SA data based on a long recall time is likely to be less reliable than data based on a short recall time.

Thirdly, the Trimbos/iMTA questionnaire for costs associated with psychiatric illness, “Treatment Inventory of Costs in Patients with psychiatric disorders” (TiC-P), which was used in 8 out of 11 studies as the method of self-reporting SA, has been validated for a recall period of 2 weeks [46]. The shortest recall time that was reported in the studies reviewed here was 4 weeks, and some studies described recall times of up to 12 months (Table I). However, one of the included studies argued that a self-report questionnaire at 6 months can pick up 85% of diary-recorded non-hospital costs [47], although the validity of this claim is hard to assess, as this study did not cover SA as a cost item. We based our estimate of the accuracy of self-reports on a previous study [48]. This latter study indicated that self-reported SA with recall periods of up to 4 weeks may provide 80–90% accurate data compared with registry data; and self-reporting accuracy may drop to 50% when recall periods extend beyond 2 months. This led us to conclude that the means of collecting SA data were highly variable, that there was a moderate–high risk of inaccuracy in most studies, and that none of the studies used the TiC-P in the way for which it was designed and validated (with a 2-week recall time).

A further limitation of the review is the possibility of missing relevant studies published in languages other than English. Only one abstract in this review was excluded from full text screening due to the article being published in another language. Therefore, it is reasonable to assume that this limitation is minor considering that one or more databases tend to post study abstract translations in English.

In conclusion, based on the studies reviewed here, it seems unlikely that the provision and delivery of an iCBT intervention as defined in this review will be a sufficient measure to reduce SA among people with CMDs. Mhealth, e-health, telehealth and other remote modes of patient treatment have gained increased significance in the context of the coronavirus disease 2019 (COVID-19) pandemic and there is a pressing need to investigate and test ways of combining technology such as the internet with treatments such as CBT. Rigorous studies (and subsequently reviews of studies) of the impact of iCBT on employment status and on absence from school or education are needed. Also, we need to improve our understanding about how iCBT impacts on the functioning of an individual in their various social, family, and community contexts. It is important to be mindful that our study exclusion criteria meant that the review did not include substance abuse studies comorbid with common mental disorders. We recognize the importance of this omission and that further research is required into SA in the relation to substance abuse and its set of interrelated social, behavioral, economic, and occupational factors. Further research is needed, too, to clarify the mechanisms of action in occupational mental health interventions (e.g., workplace services, mental ill-health treatments, peer group sessions, physical exercise, and medication) in order to design effective, tailored programs that include iCBT as an individual or integrated mode of addressing SA specifically. There remains a need for trials of iCBT (that include a process evaluation and an economic appraisal) in order to address the paucity of rigorous studies that address this outcome of SA. Improving the reliability and validity of the assessment of SA data would inform discussion beyond days of SA and decisions about the efficient distribution of resources in occupational health care between rehabilitation of employees with common mental disorders already on long-term sick-leave [10], and preventive, low-threshold, and right-on-time clinical treatments such as iCBT [18]. Ultimately, research efforts that are directed towards producing standardized, accurate and repeatable recording of SA among people with common mental disorders will further our understanding about the impact of working and the work environment on mental health.

## Supplemental Material

sj-docx-1-sjp-10.1177_14034948221075016 – Supplemental material for Internet-delivered cognitive behavioral therapy (iCBT) for common mental disorders and subsequent sickness absence: a systematic review and meta-analysis Click here for additional data file. Supplemental material, sj-docx-1-sjp-10.1177_14034948221075016 for Internet-delivered cognitive behavioral therapy (iCBT) for common mental disorders and subsequent sickness absence: a systematic review and meta-analysis by Lina Udd-granat, Jouni Lahti, Michael Donnelly, Charlene Treanor, Sami P. Pirkola, Tea Lallukka and Anne Kouvonen in Scandinavian Journal of Public Health

sj-docx-2-sjp-10.1177_14034948221075016 – Supplemental material for Internet-delivered cognitive behavioral therapy (iCBT) for common mental disorders and subsequent sickness absence: a systematic review and meta-analysis Click here for additional data file. Supplemental material, sj-docx-2-sjp-10.1177_14034948221075016 for Internet-delivered cognitive behavioral therapy (iCBT) for common mental disorders and subsequent sickness absence: a systematic review and meta-analysis by Lina Udd-granat, Jouni Lahti, Michael Donnelly, Charlene Treanor, Sami P. Pirkola, Tea Lallukka and Anne Kouvonen in Scandinavian Journal of Public Health

sj-docx-3-sjp-10.1177_14034948221075016 – Supplemental material for Internet-delivered cognitive behavioral therapy (iCBT) for common mental disorders and subsequent sickness absence: a systematic review and meta-analysis Click here for additional data file. Supplemental material, sj-docx-3-sjp-10.1177_14034948221075016 for Internet-delivered cognitive behavioral therapy (iCBT) for common mental disorders and subsequent sickness absence: a systematic review and meta-analysis by Lina Udd-granat, Jouni Lahti, Michael Donnelly, Charlene Treanor, Sami P. Pirkola, Tea Lallukka and Anne Kouvonen in Scandinavian Journal of Public Health

sj-docx-4-sjp-10.1177_14034948221075016 – Supplemental material for Internet-delivered cognitive behavioral therapy (iCBT) for common mental disorders and subsequent sickness absence: a systematic review and meta-analysis Click here for additional data file. Supplemental material, sj-docx-4-sjp-10.1177_14034948221075016 for Internet-delivered cognitive behavioral therapy (iCBT) for common mental disorders and subsequent sickness absence: a systematic review and meta-analysis by Lina Udd-granat, Jouni Lahti, Michael Donnelly, Charlene Treanor, Sami P. Pirkola, Tea Lallukka and Anne Kouvonen in Scandinavian Journal of Public Health
