# Supplementary material for: Internet-delivered cognitive behavioral therapy (iCBT) for common mental disorders and subsequent sickness absence: a systematic review and meta-analysis
Source: Scand J Public Health. 2022 Feb 4;51(1):137–47. doi: 10.1177/14034948221075016 (PMC9903245; doi:10.1177/14034948221075016)
Supplement: sj-docx-1-sjp-10.1177_14034948221075016 – Supplemental material for Internet-delivered cognitive behavioral therapy (iCBT) for common mental disorders and subsequent sickness absence: a systematic review and meta-analysis [file sj-docx-1-sjp-10.1177_14034948221075016.docx]

**Table 3A** [Alternative to Figure 2]

**Study treatment treatment treatment control control control Cohen’s d CI 95%**

**N mean SD N mean SD**

Beiwinkel et al. 2017 100 24.65 3.8 80 24.04 4.36 0.15 -0.14—0.44

Buntrock et al. 2017 202 1475 2498 204 1172 2209 0.13 -0.07—0.32

Ebert et al. 2016 132 7.32 14.71 131 5.17 10.52 0.17 -0.07—0.41

Geraedts et al. 2014 116 7.3 25.6 115 6.9 23.3 0.02 -0.24—0.27

Hange et al. 2017 46 13.4 42.6 31 8.6 33.2 0.12 -0.33—0.58

Heber et al. 2016 132 3.64 6.7 132 5.23 12.1 -0.16 -0.40—0.08

Kraepelien et al. 2018 317 2129.54 6716.21 312 1626.46 3926.78 0.09 -0.07—0.25

Persson-Asplund et al. 2018 59 3.5 2.32 58 4.25 0.96 -0.42 -0.79— -0.05

Romero-Sanchiz et al. 2017 96 250.36 2852.59 102 262.41 1375.4 -0.01 -0.28—0.27

Thiart et al. 2015 64 2.42 5.52 64 3.42 11.62 -0.11 -0.46—0.24

Overall 0.03 -0.06—0.12

Heterogeneity I^2^ =20.93%

**Table 3B**

**Study treatment treatment treatment control control control Cohen’s d CI 95%**

**N mean SD N mean SD**

Geraedts et al. 2014 116 0.4 1 115 1.6 4.9 -0.34 -0.60—0.08

Hange et al. 2017 46 16.5 33 31 21.7 65.4 -0.11 -0.56—0.35

Lindsäter et al. 2019 49 438 929 48 624 1276 -0.17 -0.57—0.23

Persson-Asplund et al. 2018 59 4.43 3.69 58 2.7 1.95 0.58 0.21—0.95

Overall -0.01 -0.42—0.40

Heterogeneity I^2^ = 80.40%

**Table 3C**

**Study treatment treatment treatment control control control Cohen’s d CI 95%**

**N mean SD N mean SD**

Beiwinkel et al. 2017 100 24.65 3.8 80 24.04 4.36 0.15 -0.14—0.44

Buntrock et al. 2017 119 2.95 6.69 129 2.22 4.95 0.12 -0.12—0.37

Geraedts et al. 2014 116 7.3 25.6 115 6.9 23.2 0.02 -0.24—0.27

Hange et al. 2017 46 16 36.7 31 19 43.4 -0.08 -0.53—0.38

Overall 0.08 -0.07—0.22

Heterogeneity I^2^ = 0.00%

**Study treatment treatment treatment control control control Cohen’s d CI 95%**

**N mean SD N mean SD**

Buntrock et al. 2017 202 1475 2498 204 1172 2209 0.13 -0.07—0.32

Geraedts et al. 2014 116 0.4 1 115 1.6 4.9 -0.34 -0.60— -0.08

Hange et al. 2017 46 13.4 42.6 31 8.6 33.2 0.12 -0.33 –0.58

Kraepelien et al. 2018 317 2129.54 6716.21 312 1626.46 3926.78 0.09 -0.07—0.25

Romero-Sanchiz et al. 2017 96 250.36 2852.59 102 262.41 1375.4 -0.01 -0.28—0.27

Overall 0.00 -0.17—0.18

Heterogeneity I^2^ = 61.21%
