# Supplementary material for: Internet-delivered cognitive behavioral therapy (iCBT) for common mental disorders and subsequent sickness absence: a systematic review and meta-analysis
Source: Scand J Public Health. 2022 Feb 4;51(1):137–47. doi: 10.1177/14034948221075016 (PMC9903245; doi:10.1177/14034948221075016)

#
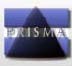
PRISMA 2009 Checklist

| **Section/topic** | **#** | **Checklist item** | **Reported on page #** |
| --- | --- | --- | --- |
| **TITLE** | | |  |
| Title | 1 | *Internet-delivered cognitive behavioral therapy (iCBT) for common mental disorders and sickness absence: a systematic review* |  |
| **ABSTRACT** | | |  |
| Structured summary | 2 | *Mental disorders are a predominant cause of sickness absence. Internet-based therapies to treat common mental disorders offer potential alternatives to face-to-face therapies due to being accessible from almost anywhere and generating cost-savings in terms of infrastructure investments and human resources.*  *The objective of this review was to review the available information on the effectiveness of internet-based interventions fro common mental disorders in terms of reducing sickness absence. Data was sourced from studies searched through PubMed-Medline, Cochrane Library, CinAhl, Embase and PsycINFO, as well as from reference lists of included studies and reviews (see hand-search in PRISMA flowchart).*  *Studies were included if they were controlled trials that compared therapist-guided internet –delivered Cognitive Behavioral Therapy (iCBT) to waitlist control, information control, care as usual, or placebo. The trials had to comprise a working-age adult (18-64 years old) patient sample, and be written in English. Studies were included if they identified sufferers of clinically determined common mental health conditions, as well as sleep disorders, in particular insomnia: (Neurotic disorders (WHO International Classification of Disease, version 10 (ICD10) codes F40- F42), depressive disorders (ICD-10 codes F32-39), Adjustment disorders F43.2, Insomnia (F51.0). In addition, we included studies of adults with self-reported mental illness if they meet the definition of a ‘case’ as defined according to an empirically verified cut point on a validated scale.*  *Also, eligible studies had to report data on sickness absence in the time interval after the intervention. The search s*coped also for *other outcomes, such as work ability, work satisfaction and work functioning, unemployment, but for the meta-analysis we had pre-decided to focus on amount of absence from work for any health related reason in the interval of the study follow-up.*  *Studies were appraised with the Cochrane Risk of Bias1 tool, and limitations of the available data were further addressed by an estimation of accuracy of the outcome measurements and of missing outcome data. The data was presented as a meta-analysis of all study timepoints and as tabulated normalized mean monthly sickness absences at the different measurement timepoints of the studies. There was no effect of iCBT on sickness absence as compared to the control setting as the total SMD was close to zero and the confidence interval extended both to favoring intervention and control.*  *The results of this study in comparison with previous reviews and the limitations of the study are discussed in the review text. The results suggest further interventions in addition to therapist guided online CBT modules need to be applied in situations where the aim is to reduce sickness absence. The limitations of the currently available data suggest further studies assessing sickness absence to be welcome, and also follow-up review of the subject, perhaps also including mobile applications, which based on a general view from the search performed for this review often* |  |


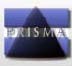
**PRISMA 2009 Checklist**

|  |  | *feature non-human guidance (automated guidance or guidance based on artificial intelligence).* |  |
| --- | --- | --- | --- |
| **INTRODUCTION** | | |  |
| Rationale | 3 | *Internet-delivery/online access to CBT therapy is relatively new, but efficacy has been documented well in other reviews. The effect on occupational performance, and in this case absence or presence at work had not yet been systematically reviewed at the time this review was planned, and remains a novel subject for common mental disorders other than depression.* |  |
| Objectives | 4 | *Our objective was to determine the effects on sickness absence of any cause, of guided online interventions based on Cognitive Behavioral therapy (iCBT), compared to treatment as usual, waitlists, enhanced treatment as usual, self- guided manuals or alternatively in comparison with face-to-face CBT, in a (predominantly) working-age population.* |  |
| **METHODS** | | |  |
| Protocol and registration | 5 | *PROSPERO 2020 CRD42020151604 Available from: https*[*://www.crd.york.ac.uk/prospero/display_record.php?ID=CRD42020151604*](http://www.crd.york.ac.uk/prospero/display_record.php?ID=CRD42020151604) |  |
| Eligibility criteria | 6 | *Study characteristics Participants: mainly adults (18-65). Interventions: therapist guided online interventions described as fully or partly based on Cognitive Behavioral therapy. Comparisons: treatment as usual, waitlist, enhanced treatment as usual, self-guided manuals. Alternative comparison with face-to-face CBT. Outcomes: sickness absence after the intervention and study design: randomized clinical trials (RTCTs). Also nonrandomized trials were mentioned in the protocol, but as a substantial number of RCTs were retrieved, we decided to deviate from the protocol at this point.*  *Report characteristics: only published studies in English were considered (due to time and resource constraints). No year limits were set for the studies selected, but as the technology for iCBT is relatively new, the studies analysed in fulltext had all been performed within a narrow time frame of 1-2 decades.* |  |
| Information sources | 7 | *Databases searched: Pubmed-MEDLINE (coverage 1966-present, last searched 11/2020), OVID-PsycINFO (coverage 1967-present, last searched 11/2020) , EBSCO-CINAHL (coverage 1937-present, last searched 11/2020), Embase (coverage 1966-present, last searched 11/2020), Cochrane (CENTRAL) (coverage 1992-present, last search 11/2020).*  *Contact with study authors for fulltext articles for Elin Lindsäter "Internet-Based Cognitive Behavioral Therapy for Chronic Stress: A Randomized Controlled Trial" (Psychother Psychosom. 2018;87(5):296-305.), Per Carlbring, "Randomised controlled non-inferiority trial with 3-year follow-up of Internet-delivered versus face-to-face group cognitive behavioural therapy for depression" (Journal of Affective Disorders, 2013)Virginia Plummer "A WeChat- Based "Three Good Things" Positive Psychotherapy for the Improvement of Job Performance and Self-efficacy in Nurses with Burnout symptoms: A Randomized Controlled Trial" (Journal of Nursing Management, 2019 and 2020 ) , Caroline Bell "Effectiveness of computerised cognitive behaviour therapy for anxiety disorders in secondary care" (The Australian and New Zealand journal of psychiatry 07/ 2012, not retrieved), Kate Cavanagh, "The effectiveness of computerized cognitive behavioural therapy in routine care"( Br J Clin Psychol, 2006, not retrieved),Lasse Sander,*  *"Effectiveness of a Guided Web-Based Self-help Intervention to Prevent Depression in Patients With Persistent Back* |  |


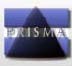
**PRISMA 2009 Checklist**

|  |  | *Pain: The PROD-BP Randomized Clinical Trial" (JAMA Psychiatry Oct 2020), Kelly Schaffer "Effects of an Internet- Based Cognitive Behavioral Therapy for Insomnia Program on Work Productivity: A Secondary Analysis." ( Annals of behavioral medicine, 2020), Kaia Kjørstad, "The Effect of Reducing Insomnia Severity on Work- and Activity-Related Impairment" (Behav. Sleep Med. 2020) Gavin Andrews, "Clinician-assisted Internet-based treatment is effective for panic: A randomized controlled trial" (The Australian and New Zealand journal of psychiatry 07/ 2010, not retrieved), Thomas Berger, "Evaluating an e-mental health program (“deprexis”) as adjunctive treatment tool in psychotherapy for depression: Results of a pragmatic randomized controlled trial" (Journal of Affective Disorders, 2018)* |  |
| --- | --- | --- | --- |
| Search | 8 | *The Pubmed-Medline search was structured as follows:*  *#1 iCBT OR i‐CBT OR ePsych* OR e‐Psych* OR cCBT OR c‐CBT*  *#2cognitive behavioral therapy [MH] OR behavior therapy [MH] OR "cognitive therapy" OR "cognitive behavio*" OR psychotherap* OR "psychological therap*"*  *#3 ("rational emotive*" OR "problem sol*" OR "problem focus*" OR "solution focus*" OR psychoeducat* OR psycho‐educat* OR psychodrama OR psycho‐drama* OR mindfulness* OR "anxiety management" OR "stress management" OR “guided imagery” ) AND (therap* OR train* OR defusion OR modif* OR restructur* OR technique* OR intervention OR treatment*)*  *#4telemedicine OR "technology based" OR telecomm* OR blog* OR "cell phone" OR cellphone OR "chat room" OR computer* OR digital OR ehealth OR e-health OR "electronic health" OR e-mail* OR email* OR e-portal OR portal OR eTherap* OR e-therap* OR "information technology" OR messaging OR internet [TW] [MH] OR internet-delivered OR internet-based OR "portable tablet" OR ipad OR i-pad OR podcasts OR "smart phone" OR smartphone OR "social network site" OR mhealth OR m-health OR mobile OR multi-media OR multimedia OR online OR on-line OR SMS OR "social media" OR software OR telecomm* OR telehealth* OR telemed* OR telemonitor* OR telepsych* OR teletherap* OR tele-health* OR tele-med* OR tele-monitor* OR tele-psych* OR tele-therap* OR "text message" OR virtual* OR web OR web-based OR WWW OR videoconferencing OR "video conference" OR webcast* OR "wireless technology" OR tele-comm* OR elearning OR "blended learning"*  *#6 (#2 OR #3) AND #4*  *#7 #1 OR #6*  *#8sick leave OR “return to work” OR “back to work” OR "out of work" OR absenteeism OR presenteeism OR "sick days" OR "illness days" OR "sick listed" OR disabled OR invalid OR disability OR disablement OR invalidity OR “workman's compensation" OR "workman compensation" OR "workmen's compensation" OR "workmen compensation" OR "worker's compensation" OR "worker compensation" OR working OR occupational OR work OR worker* OR employee* OR "Illness benefit" OR "sickness benefit" OR pension* OR "social security" OR "social insurance" OR beneficiaries*  *#9 #7 AND #8*  *In addition, for Pubmed-Medline we applied the search string suggested in the Cochrane manual for randomized controlled trials.* | Suppl. |


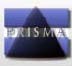
**PRISMA 2009 Checklist**

|  |  |  |  |
| --- | --- | --- | --- |
| Study selection | 9 | *Abstracts and titles were screened in the Covidence software by two independent authors (LUG, JL) and conflicts were resolved partly together with a third author (AK). After this, fulltext versions of articles were screened for eligibility also by two independent authors in the covidence format (LUG, JL) and all fulltext conflicts were resolved together with a third author (AK).*  *References of reviews and protocols deemed important were hand searched and screened (LUG) and of these, 3 fulltext articles were screened also by the second and third author (JL, AK). None of the hand-searched articles were included in the final study.*  *References were fused into studies based on trial ID:s, except for the Kaldo et al 2018. reference that was fused with references by Hallgren et al, 2015 and Kraepelien et al. 2019 based on study similarity and based on that the trial ID by Kaldo et al. German trial register entry refers to a publication with the Swedish register trial ID of Hallgren et al. and Kraepelien et al.*  *i.e., screening, eligibility, included in systematic review, and, if applicable, included in the meta-analysis).* |  |
| Data collection process | 10 | *Data extraction was made with a form based on items recommended in the Cochrane manual. It was first sent out for comments to the whole research team, and then independently piloted on one study by JL and LUG and finalized in discussion with AK before use. Data extraction was performed in the Covidence software as independent extraction in duplicate by two researchers (LUG, JL) before synthesis. Additional data was sought by contacting authors (Elin Lindsäter, Claudia Buntrock, Sandra Schlicker (data not possible to retrieve), Martin Kraepelien, Viktor Kaldo (data not possible to retrieve), Maria Eriksson, Dominique Hange, Pablo Romiro-Sanchez (data not possible to retrieve), Louise Mewton and Gavin Andrews (data not possible to retrieve).* |  |
| Data items | 11 | *Study characteristics: type, year, authors and contacts, country, size, setting, funding, conflicts of interest, study aims, power calculations and conclusions*  *Patients: number at start and end, baseline characteristics (age, sex, education, employment, baseline sickness absence, other mental health treatments)*  *Intervention: description, background and references, description of guidance Control: classification (active/passive), description*  *Outcome: means of collecting outcome, means of adjusting for missing outcomes, published outcome description/details, means and sd:s for intervention and control subjects of days of sickness absence/time period at different timepoints of study and possible recalculations to retrieve these.* | Suppl. |
| Risk of bias in individual studies | 12 | *Risk of bias assessment was performed with the RoB1 tool in the Covidence software. Analysis was performed in independently and in duplicate by two authors (LUG, JL) , before resolving conflicts. Risk of bias and reliability of the sickness absence outcome data was given special emphasis in the risk assessment, and the sickness absence outcome was especially evaluated under the RoB1 topics “blinding of outcome assessment”, “other bias” and “overall risk of bias”. The risk of bias analysis is presented in graphical form in this review next to the meta-analysis. The reliability of outcome assessment in the included studies is also further presented in Table 1 and discussed in the review text.* |  |
| Summary measures | 13 | *The principal summary measure is the difference in means of sickness absence days (or costs) between intervention* |  |

|  |  | *and control groups after intervention.* |  |
| --- | --- | --- | --- |
| Synthesis of results | 14 | *Results were synthesized using the RevMan5 software. Studies were subgrouped according to disorder studied and other means of classifying the studies based on baseline levels of sickness absence is discussed in the review and the data is presented in Figure 2 as a meta-analysis and in Table 2 as normalized group means, in which both the timespan studied and the national basal level of sickness absence has been taken into consideration. The overall I2 suggested the studies consistent enough for meta-analysis.* |  |

Page 1 of 2

| **Section/topic** | **#** | **Checklist item** | **Reported on page #** |
| --- | --- | --- | --- |
| Risk of bias across studies | 15 | *Measurements of sickness absence have not been a primary outcome for most studies, and power analysis/sample size calculations has not been done to achieve power for this end. The means of collecting sickness absence data is highly variable, and there is a moderate-high risk of inaccuracy in most studies. Register data was used only in one study and none of the studies used self reporting with TiC-P in the way it was designed and validated (with a 2-week recall time).* |  |
| Additional analyses | 16 | *The study protocol did not pre-specify what further analysis we intended to do with the material, as we were unsure of whether a large enough number of studies would be retrieved to perform quantitative analysis.*  *The variation in presentation of sickness absence outcome prompted us to attempt normalization of this outcome to timespans and population background levels of sickness absence (Table2) alongside conventional meta-analysis, to allow for more intuitive comparison between studies.*  *Upon decision of the final set of studies for inclusion we decided to subgroup the studies according to diagnostic groups into “depression studies” and “other disorder studies*” *based on the clinical relevancy of such a divide. The prevention-vs. treatment approach in the studies and highly different baseline levels of sickness absence in the studies also prompted us to examine this perspective as subgroup analysis.* |  |
| **RESULTS** | | |  |
| Study selection | 17 | *2831 titles and abstracts were screened based on inclusion and exclusion criteria, 68 records were screened in full text, and 20 records included as 11 studies in the review. Please see the PRISMA flow-chart attached.* | Suppl |
| Study characteristics | 18 | *Please see the supplementary table characteristics of studies. For legibility, the table does not include all data charted.* |  |
| Risk of bias within studies | 19 | *Risk of bias was assessed by two independent authors (LUG, JL) with the RoB1-tool in the Covidence environment. Conflicts were resolved by discussion after completion of each separate assessment and upon finalization of the assessment of all studies. Please see the risk of bias assessment in Figure 1A and the summary of sickness absence outcome-related bias assessment in Table1.* |  |
| Results of individual studies | 20 | *Sickness absence outcome effect sizes were presented as standardized mean differences as recently recommended in a Cochrane review of sickness absence interventions in depression (Nieuwenhuijsen, Verbeek et al. 2020).* |  |
| Synthesis of results | 21 | *Please see Figure 2 and supplementary Figures for the data synthesis.* |  |

| Risk of bias across studies | 22 | *Please see Figure 1B for the funnel plot. It was not visibly skewed, suggesting no major risk of publication bias.* |  |
| --- | --- | --- | --- |
| Additional analysis | 23 | *The subgroup analyses were performed as planned upon completion of the fulltext assessment (please see above), despite that the data gathering and subsequent analysis revealed a “no-change”- overall effect. Analysis was repeated also with only studies fulfilling the moderate quality criteria in Table 1, which did not change the outcome, as described in the review. Subgroup analysis for depression can be seen in Figure 2B, and subgroup analyses based on baseline sickness absence in the Supplementary Figure “High and Low baseline sickness absence”. We decided not present the “other disorders” subgroup analysis, as this group was statistically more heterogeneous, and would not have added any clinical significance as compared to the analysis of all studies combined.* |  |
| **DISCUSSION i** | | |  |
| Summary of evidence | 24 | *The main finding of this study is that sickness absence ompng patients with common mental disorders was not affected by therapist-guided multimedia module-type iCBT. A secondary finding was that sickness absence was both recorded and reported in very different ways in the studies of iCBT for common mental disorder, underlining the need to develop consensus and guidelines for these procedures.* |  |
| Limitations | 25 | *The limitations of the studies and the data reported is discussed in the review text. We retrieved almost all the data identified in the literature search, exceptions being: the data from a previous individual data meta analysis based on sickness absence outcomes from studies applying the Sheehan disability index (Mackenzie et al. 2014) had to be left out due to the aggregate data set representing both guided and unguided interventions in combination, without the possibility to separate the two settings (confirmed by communication with study authors). The standard deviation of the mean sickness absence costs had to be a best estimate (based on both absenteeism and presenteeism) for Romero-Sanchiz et al. 2017. Schlicker et al. 2020 described collection of sickness absence data, but published data was too fragmentary for analysis. Communication with authors confirmed that no further data was available on the subject from this study.* |  |
| Conclusions | 26 | *The results imply that when aiming to reduce sickness absence, providing an iCBT intervention as defined and selected for in this review will probably not be a sufficient measure. Further research is needed to clarify what activating measures, or other, unrelated interventions (like workplace interventions, peer group sessions, physical exercise, medications etc.) should be combined with the iCBT program to specifically target also sickness absence.* |  |
| **FUNDING** | | |  |
| Funding | 27 | *Anne Kouvonen has received funding for the initiation and managing of the project from the Academy of*  *Finland (grant 315343) and from the Strategic Research Council at the Academy of Finland (grants 327145 and 327148 for the DigiIn Project). Lina Udd-Granat has been partly financed by Occupational Health Helsinki. None of the authors have conflicting financial interests in the study.* |  |

*From:* Moher D, Liberati A, Tetzlaff J, Altman DG, The PRISMA Group (2009). Preferred Reporting Items for Systematic Reviews and Meta-Analyses: The PRISMA Statement. PLoS Med 6(7): e1000097. doi:10.1371/journal.pmed1000097

For more information, visit: [**www.prisma-statement.org**.](http://www.prisma-statement.org/)

**All search strings**

Page 2 of 2

**Pubmed-Medline search rerun 6.11.2020 (please see above main checklist)**

**Cochrane CENTRAL search Date Run: 06/11/2020 16:23:53**

| ID #1  #2  #3  #4  #5  #6 | Search Hits iCBT 576  i-CBT 289  cCBT 148  c-CBT 91  e-Psych* 127  #1 or #2 or #3 or #4 #5 | 743 | |
| --- | --- | --- | --- |
| #7 | MeSH descriptor: [Cognitive Behavioral Therapy] explode all trees | | 8934 |
| #8  #9  #10  #11  #12  #13  #14  #15  #16  #17  #18  #19  #20  #21  #22  #23  #24  #25  #26  #27  #28  #29  #30 | MeSH descriptor: [Psychotherapy, Rational-Emotive] explode all trees MeSH descriptor: [Psychodrama] this term only 31  MeSH descriptor: [Mindfulness] this term only 850  "anxiety management" 290  "stress management" 2662 therapy695060 intervention 367682  treatment 766345  #11 or #12 2928  #13 or #14 or #15 1142701  #16 and #17 2581  #7 or #8 or #9 or #10 or #18 11272  MeSH descriptor: [Telemedicine] explode all trees 2547  "technology based" 624  MeSH descriptor: [Cell Phone] explode all trees 1354  blog* 285  "online intervention" 642  MeSH descriptor: [Patient Portals] explode all trees 17  MeSH descriptor: [Mobile Applications] explode all trees 640  eTherapy 31  e-Therapy 195  multimedia 1397  sms 2686 | | 26 |

| #31  #32 | "social media" 1322  MeSH descriptor: [Videoconferencing] this term only 180 |  |
| --- | --- | --- |
| #33 | webcast 71 |  |
| #34 | elearning 712 |  |
| #35 | "blended Learning" 109 |  |
| #36 | #20 or #21 or #22 or #23 or #24 or #25 or #26 or #27 or #28 or #29 or #30 or #31 or #32 or #33 or #34 or #35 | 11203 |
| #37 | #19 and #36 564 |  |
| #38 | #6 or #37 1247 |  |
| #39 | MeSH descriptor: [Sick Leave] explode all trees 554 |  |
| #40 | "return to work" 2404 |  |
| #41 | "back to work" 81 |  |
| #42 | "out of work" 52 |  |
| #43 | MeSH descriptor: [Workers' Compensation] explode all trees 46 |  |
| #44 | absenteeism 1939 |  |
| #45 | presenteeism 371 |  |
| #46 | MeSH descriptor: [Pensions] explode all trees 24 |  |
| #47 | employee 1838 |  |
| #48 | "occupational disability" 27 |  |
| #49 | "occupational disablement" 0 |  |
| #50 | "occupational invalidity" 0 |  |
| #51 | "work invalidity" 0 |  |
| #52 | #39 or #40 or #41 or #42 or #43 or #44 or #45 or #46 or #47 or #48 or #49 or #50 or #51 6281 |  |
| #53 | #38 and #52 38 |  |

**Embase search run 18.11.2020**

1. (iCBT or i-CBT or ePsych* or e-Psych or cCBT or c-CBT).mp. [mp=title, abstract, heading word, drug trade name, original title, device manufacturer, drug manufacturer, device trade name, keyword, floating subheading word, candidate term word]
2. cognitive behavioral therapy/
3. behavior therapy/
4. "cognitive therapy".mp. [mp=title, abstract, heading word, drug trade name, original title, device manufacturer, drug manufacturer, device trade name, keyword, floating subheading word, candidate term word]
5. "cognitive behavio*".mp. [mp=title, abstract, heading word, drug trade name, original title, device manufacturer, drug manufacturer, device trade name, keyword, floating subheading word, candidate term word]
6. psychotherap*.mp. [mp=title, abstract, heading word, drug trade name, original title, device manufacturer, drug manufacturer, device trade name, keyword, floating subheading word, candidate term word]
7. "psychological therapy*".mp. [mp=title, abstract, heading word, drug trade name, original title, device manufacturer, drug manufacturer, device trade name, keyword, floating subheading word, candidate term word]
8. 2 or 3 or 4 or 5 or 6 or 7
9. ("rational emotive*" or "problem sol*" or "problem focus*" or "solution focus*" or psychoeducat* or psycho-educat* or psychodrama or psycho- drama* or mindfulness* or "anxiety management" or "stress management" or "guided imagery").mp. [mp=title, abstract, heading word, drug trade name, original title, device manufacturer, drug manufacturer, device trade name, keyword, floating subheading word, candidate term word]
10. (therap* or train* or defusion or modif* or restructur* or technique* or intervention or treatment*).mp. [mp=title, abstract, heading word, drug trade name, original title, device manufacturer, drug manufacturer, device trade name, keyword, floating subheading word, candidate term word]
11. ("technology based" or telecomm* or blog* or "cell phone" or cellphone or "chat room" or computer* or digital or ehealth or e-health or "electronic health" or e-portal or portal or eTherap* or e-therap* or "information technology" or messaging or internet or internet-delivered or internet-based or "portable tablet" or ipad or i-pad or podcasts or "smart phone" or smartphone or "social network site" or mhealth or m-health or mobile or multi-media or multimedia or online or on-line or SMS or "social media" or telecomm* or telehealth* or telemed* or telemonitor* or telepsych* or teletherap* or tele-health* or tele-med* or tele-monitor* or tele-psych* or tele-therap* or "text message" or virtual* or web or web-based or videoconferencing or "video conference" or webcast* or "wireless technology" or tele-comm* or elearning or "blended learning").mp. [mp=title, abstract, heading word, drug trade name, original title, device manufacturer, drug manufacturer, device trade name, keyword, floating subheading word, candidate term word]
12. telemedicine/
13. sick leave/
14. absenteeism/
15. return to work/
16. back to work.mp.
17. out of work.mp.
18. presenteeism/
19. sick days.mp.
20. illness days.mp.
21. sick listed.mp.
22. workman compensation/
23. workman's compensation.mp.
24. workmen compensation.mp.
25. workmen's compensation.mp.
26. worker's compensation.mp.
27. worker compensation.mp.
28. occupational.mp.
29. worker/
30. employee/
31. illness benefit.mp.
32. sickness benefit.mp.
33. pension/
34. social security/
35. social insurance/
36. beneficiaries.mp.
37. 8 or 9

38. 10 or 11 or 12

39. 37 and 38

40. 1 or 39

41. 13 or 14 or 15 or 16 or 17 or 18 or 19 or 20 or 21 or 22 or 23 or 24 or 25 or 26 or 27 or 28 or 29 or 30 or 31 or 32 or 33 or 34 or 35 or 36

42. 40 and 41

43. limit 42 to exclude medline journals

**OVID-PSycInfo searchstring 06112020**

1. Computer Assisted Therapy/ or Online Therapy/ or iCBT.mp.
2. i-cbt.mp.
3. ccbt.mp.
4. c-CBT.mp.
5. Digital Interventions/ or etherapy.mp.
6. 1 or 2 or 3 or 4 or 5
7. Cognitive Therapy/ or Cognitive Behavior Therapy/ or Behavior Therapy/ or cognitive behavioral therapy.mp. or behavior therapy.mp.
8. psychotherapy.mp. or Psychotherapy/
9. Rational Emotive Behavior Therapy/ or rational emotive.mp.
10. ("stress management intervention" or "stress management therapy").mp.
11. ("anxiety management intervention" or "anxiety management therapy").mp.
12. 7 or 8 or 9 or 10 or 11
13. telemedicine.mp. or Telemedicine/
14. Computer Assisted Instruction/ or technology based.mp.
15. blog.mp.
16. cell phone.mp. or Mobile Phones/
17. email.mp. or Computer Mediated Communication/ or online.mp.
18. ipad.mp. or Tablet Computers/
19. Mobile Health/ or m-health.mp.
20. podcast.mp.
21. (online or internet).mp. or Internet/
22. telehealth.mp.
23. web-based.mp.
24. Teleconferencing/
25. Telepsychology/
26. Videoconferencing/
27. Electronic Communication/ or wireless.mp.
28. Electronic Learning/ or elearning.mp.

29 12 or 13 or 14 or 16 or 17 or 18 or 19 or 20 or 21 or 22 or 23 or 24 or 25 or 26 or 27 or 28

30 12 and 29

31 29 or 30

1. sick leave.mp. or Employee Leave Benefits/
2. return to work.mp. or Reemployment/
3. Employee Absenteeism/ or absenteeism.mp.
4. presenteeism.mp.
5. Employment Status/ or work disability.mp. or Vocational Rehabilitation/
6. Workers' Compensation Insurance/ or workman compensation.mp.
7. social security.mp. or Social Security/
8. worker.mp. or employee.mp. or Personnel/ 40 32 or 33 or 34 or 35 or 36 or 37 or 38 or 39 41 31 and 40

**CinAhl search run 11.11.2020**


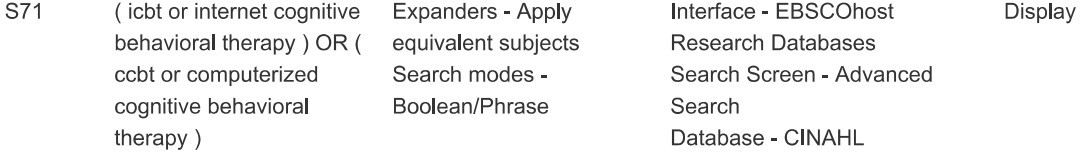


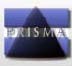
**PRISMA 2009 Checklist**


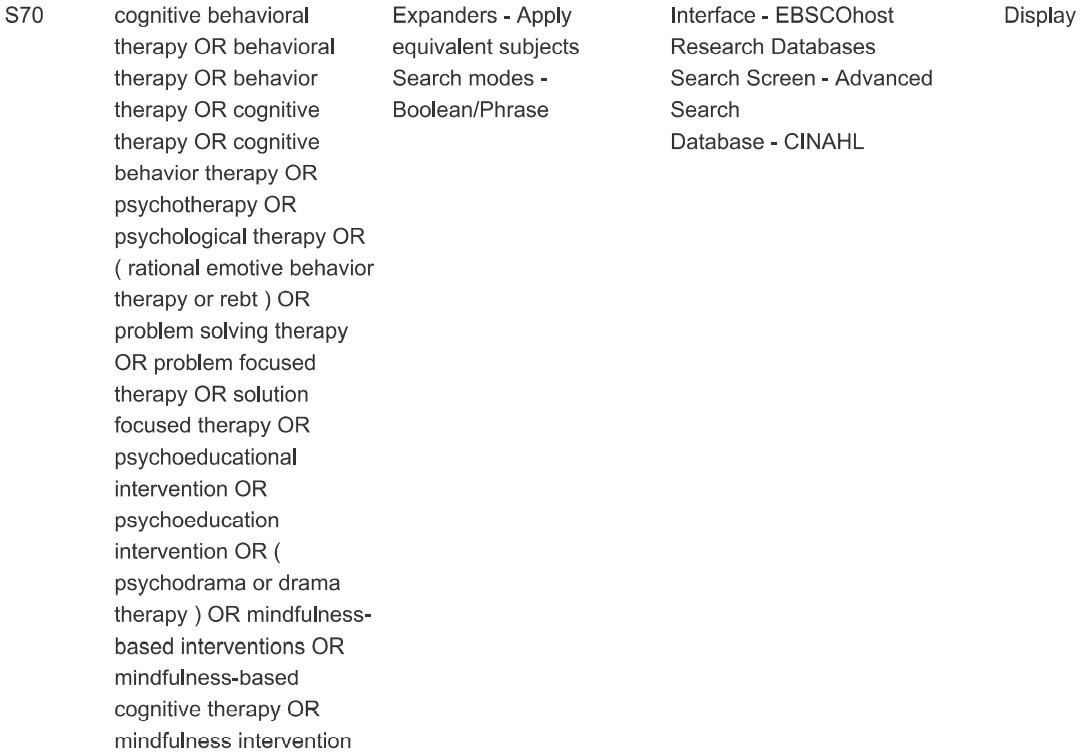

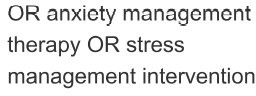


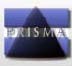
**PRISMA 2009 Checklist**


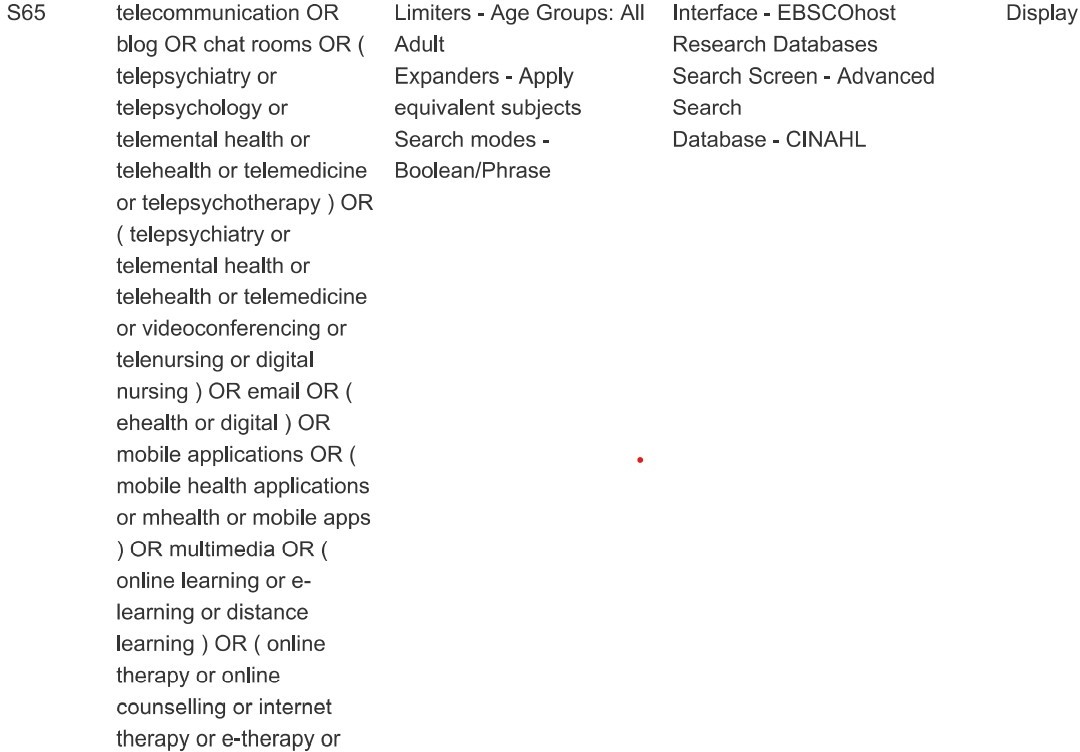


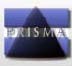
**PRISMA 2009 Checklist**


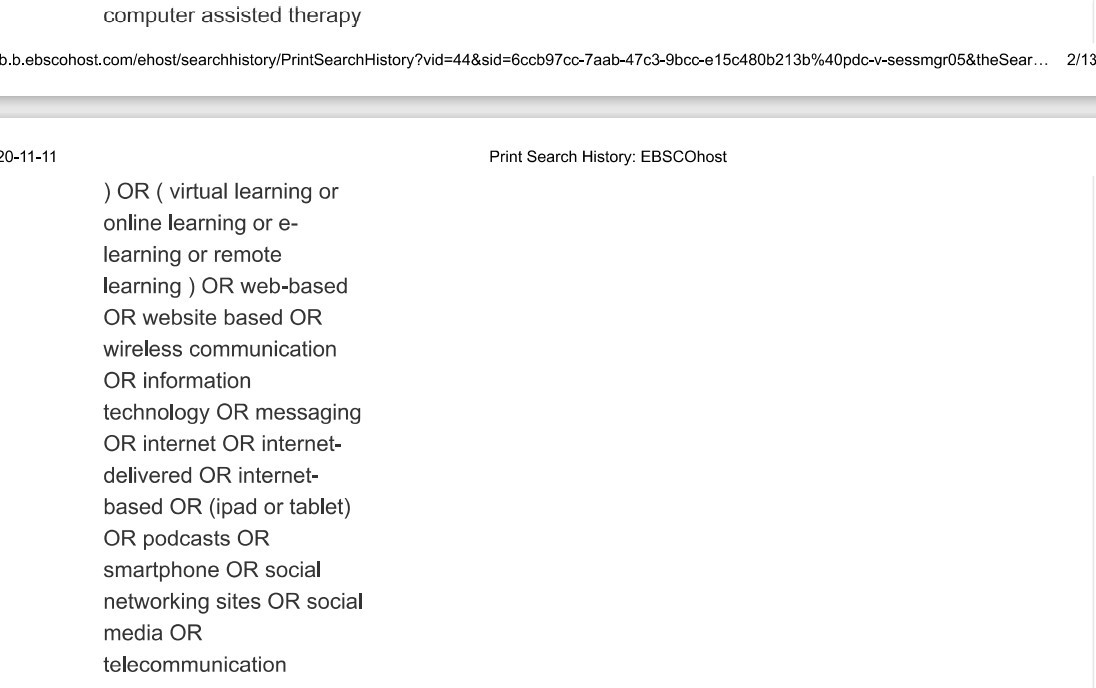

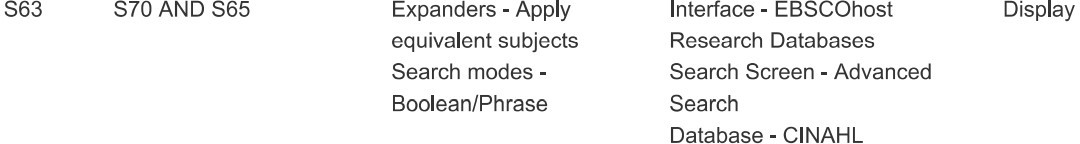


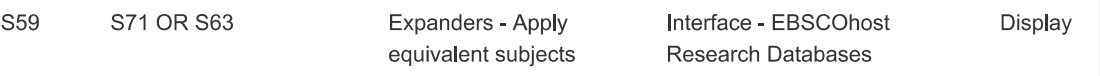


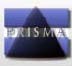
**PRISMA 2009 Checklist**


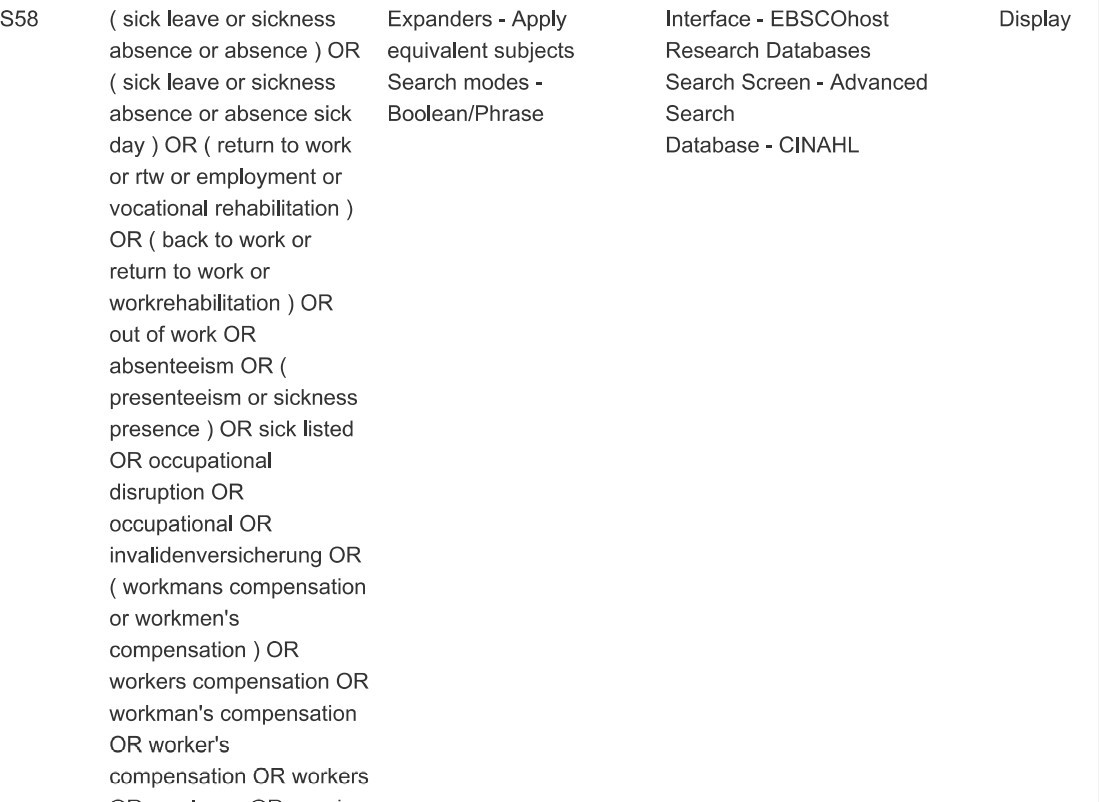

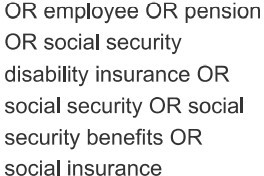


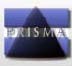
**PRISMA 2009 Checklist**


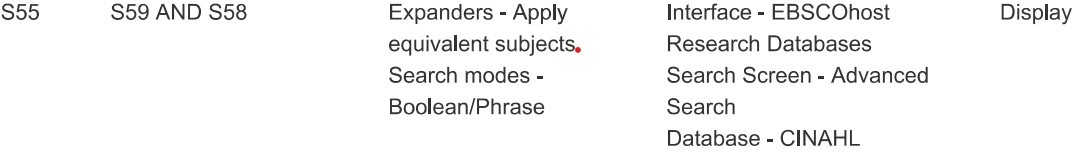

Supplement: sj-docx-2-sjp-10.1177_14034948221075016 – Supplemental material for Internet-delivered cognitive behavioral therapy (iCBT) for common mental disorders and subsequent sickness absence: a systematic review and meta-analysis [file sj-docx-2-sjp-10.1177_14034948221075016.docx]
