# Supplementary material for: Internet-delivered cognitive behavioral therapy (iCBT) for common mental disorders and subsequent sickness absence: a systematic review and meta-analysis
Source: Scand J Public Health. 2022 Feb 4;51(1):137–47. doi: 10.1177/14034948221075016 (PMC9903245; doi:10.1177/14034948221075016)
Supplement: sj-docx-3-sjp-10.1177_14034948221075016 – Supplemental material for Internet-delivered cognitive behavioral therapy (iCBT) for common mental disorders and subsequent sickness absence: a systematic review and meta-analysis [file sj-docx-3-sjp-10.1177_14034948221075016.docx]

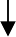

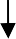

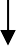

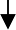


20 records assigned to 12 separate studies, one study excluded due to incomplete data

Full-text records assessed for eligibility (N = 68), conflicts (N=5, 7.7%) resolved with 3^rd^ author,

non-CBT interventions (N=2) excluded by psychiatrist

Full-text records excluded, (N = 48 )

Wrong outcomes (no sickness absence, N = 18)

Wrong type of intervention (N= 22)

-No guidance (N=14)

-Not online (N=6)

-No CBT-base (N=4)

-other reason (N=1)

Wrong participants (no CMD, N=3)

Wrong study type (no RCT report, N=2)

Records excluded (N = 2847)

Individual records screened (N = 2781) conflicts (N=114, 4.1%) resolved,

9 conflicts (0.3%) resolved with 3^rd^ author

**Included**

**Eligibility**

**Screening**


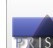
PRISMA 2009 Flow Diagram

**Identification**

Records identified through database searches (N=3090, including duplicates)

Pubmed-MEDLINE past -01/2020 (N=1613) Pubmed-MEDLINE 01-11/2020 (N=217)

OVID-PsycINFO 11/2020 (N=115) EBSCO-CINAHL 11/2020 (N=280) Embase 11/2020 (N=839)

Cochrane (CENTRAL) (N=26)

RCT- records identified through reviews and study protocols from the database search

(N = 131)

*From:* Moher D, Liberati A, Tetzlaff J, Altman DG, The PRISMA Group (2009). *P*referred *R*eporting *I*tems for *S*ystematic Reviews and *M*eta- *A*nalyses: The PRISMA Statement. PLoS Med 6(7): e1000097. doi:10.1371/journal.pmed1000097

Studies included in qualitative and quantitative synthesis (N=11)

**For more information, visit** [**www.prisma-statement.org.**](http://www.prisma-statement.org/)
