# Supplementary material for: Internet-delivered cognitive behavioral therapy (iCBT) for common mental disorders and subsequent sickness absence: a systematic review and meta-analysis
Source: Scand J Public Health. 2022 Feb 4;51(1):137–47. doi: 10.1177/14034948221075016 (PMC9903245; doi:10.1177/14034948221075016)

**Supplementary Table 1**

**Characteristics of the included studies**

**Beiwinkel et al. 2017**

**Methods** Parallel RCT in Germany, single center, recruitment via mail invitations to members of statutory health insurance company KHH

**Participants** 180 adults from general population, with high risk for sickness absence due to depression (based on previous depression, and current or previous sickness absence).

**Intervention** 3 months, 12 weekly sessions (30-45min) of interactive elements, videos, audios, illustrations, graphs, exercises and guidance. Based on CBT, awareness training and systemic counseling. Trained psychologists gave feedback upon request via telephone/email.

**Outcomes** Register data on sickness absence by the insurance company. Data presented as number of absence times, group sickness absence days means, percentages of participants absent at least once, within-group absence reductions and absence durations. Baseline data of 3 months before intervention and follow-up data between 3 to 6 months after baseline. Data from post-assessment at 3 months is not presented.

**Control intervention** wait-list with access to online text-based psychoeducation

**Primary aims and conclusions, achieved power** Aims: Efficacy of reducing depression and sickness absence. Author's conclusions: the intervention reduced depressive symptoms among adults with sickness absence. Sample size: A lower power than calculated was achieved, the final power to detect an effect of d=0.3 was 0.51.

**Further baseline characteristics of participants** Mainly employed 83,7% (I) 74% (C), mainly middle education 64,3%(I), 42%/C), mainly female 66% (I), 71.3%(C), more intervention group depression medication 53%(I), 36% (C) and previous psychotherapy 30% (I), 20% (C)

**Notes** Funded by the European Union Innovation Incubator as regional economic development support for Novego AG, developer of the intervention.

**Buntrock et al. 2017**

**Methods** Parallel RCT in Germany, single center, recruitment via advertisements, mainly through statutory health insurance company BARMER-GEK

**Participants** 406 adults from general population with subtreshold depression (CES-D 16) but not major depression or psychotherapy or psychotherapy plans in latest 6 months or at recruitment.

**Intervention** Six interactive multimedia sessions, 30 min each, performed at a pace of 1-2 sessions per week, based on psychoeducation, behavioral activation and on problem-solving therapy techniques. Option of 42 supporting text messages. Guidance after each session by health professionals and graduate students supervised by clinical psychologists.

**Outcomes** Sickness absence costs at the 12 month follow-up timepoint. Outcome was based on self-report of sickness absence days in the previous 3 months by a modified TiC-P-Questionnaire, data was collected at baseline, 6 months and 12 months.

**Control intervention** access to psychoeducational material

**Primary aims and conclusions, achieved power** Aims: Cost-efficacy and cost-utility of the intervention in depression prevention. Author conclusions: the intervention restores health in people with subtreshold depression and reduces the risk of major depression. The intervention as an acceptable likelihood of being more cost-effective than enhanced usual care. Sample size: Power for primary outcome (risk of depression) was achieved. Power calculations to detect differences in sickness absence were not done.

**Further baseline characteristics of participants** Mainly employed 84,2%(I), 80,9% (C), highly educated 81,2% (I), 81,9% (C), mainly female 73,8%(I), 74% (C), previous psychotherapy common 43.6% (I), 42.2% (C) despite 6 month limit for inclusion, antidepressants 24.8% (I), 21.6%(C)

**Notes** Some of the authors stakeholders in "Institute for Online Health Trainings" providing the intervention.

**Ebert et al. 2016**

**Methods** Same protocol as Heber et al. 2016. Parallel RCT in Germany, single center, recruitment via occupational health, mainly through statutory health insurance company BARMER-GEK

**Participants** 264 currently employed adults from general population, with scores 22 on the Perceived Stress Scale (PSS-10)

**Intervention** Eight online multimedia sessions, 45-60min each, containing text, interactive exercises, audio and video files. Performed within seven weeks. Contents based on psycho-education, problem-solving and emotional regulation techniques. Guidance: responses to exercises and reminders by psychologists and trained master’s-level psychology students

**Outcomes** Sickness absence days at baseline and at 6 month follow-up, self-reported with TiC-P, with a recall time of the latest 3months

**Control intervention** Wait-list and access to treatment as usual

**Primary aims and conclusions, achieved power** Aims: efficacy of internet-based stress management for employees. Authors’ conclusions: The intervention effectively reduces symptoms of perceived stress. No significant effects were found for absenteeism. Sample size: achieved power for efficacy of symptom decrease, no calculations for the sickness absence outcome.

**Further baseline characteristics of participants** All participants employed, mainly female 85,6% (I), 86,3%(C), mainly highly educated 71,2%(I), 72,5%(C), previous psychotherapy 27.3%(I), 31.3%(C), current psychotherapy 7.6%(I), 8.4%(C)

**Notes** Some of the authors are stakeholders of the “Institute for Online Health Training" providing the intervention.

**Geraedts et al. 2014**

**Methods** Parallel RCT in the Netherlands, single center, recruitment via advertisements, and self-enrollment: advertisement posters and information on the intranet sites of six Dutch companies.

**Participants** 231 employees from these six companies, with depression symptoms (CES-D 16), who were not currently on (partial/full) sick leave.

**Intervention** Six weekly lessons with assignments. (One week extra time given in case of delay). Lessons based on problem solving treatment and cognitive therapy. Online feedback given after each completed assignment by trained master-level students in clinical psychology, all feedback also reviewed by first author prior to distribution.

**Outcomes** The study reports self-reported sickness absenteeism in days. A modified version of TiC-P was used, where recall periods differed per assessment: 3 months (t0), 8 weeks (t1), 4 months (t2), 6 months (t3).

**Control intervention** Care as usual (participants recieved an e-mail with the advice to consult their occupational physician or a psychologist if they wanted treatment for their depressive symptoms).

**Primary aims and conclusions, achieved power** Aims: efficacy and cost-efficacy of intervention on symptoms of depression of employees not on sick-leave Authors’ Conclusions: the intervention Happy@Work is not more effective in reducing depressive symptoms than a control treatment over the period of 1 year. Power analysis: Sample size for efficacy outcomes reached (set at 200) no power analysis for absenteeism

**Further baseline characteristics of participants** All participants employed, mean age 43(I), 43.8(C), slight majority females 66.4%(I), 58.3%(C), Education high 63.8% (I), 63.5% (C) or middle 26.7% (I), 32.2% (C). Of all participants 6% had used antidepressive medication and 12.1% psychotherapy.

**Notes** No conflicts of interest declared.

**Hange et al. 2017**

**Methods** Parallel RCT in Sweden, multicentre, 16 recruiting primary care centres

**Participants** 77 adult patients attending primary care, with mild-moderate depression according to DSM-IV (assessed viaMINI), MADRS-S <35, and with interest in iCBT.

**Intervention** Seven online modules, performed in 8 - 12 weeks, containing texts, audio and video material, based on behavioral activation and components of acceptance and commitment therapy. Weekly therapist e-mail or telephone support, (a minimum of three telephone contacts during intervention), plus additional contact upon request.

**Outcomes** Percentage of participants on (any number of days of) sick leave, mean number of sickness absence days among these participants. Sickness absence data collected by self-report for the months 0-3 and 4-6, 7-12 and at baseline (past 12 months).

**Control intervention** Treatment as usual (by the end of the study 39% of control group participants were recieving face- to- face psychotherapy)

**Primary aims and conclusions, achieved power** Aims: effects of the intervention on depression and quality of life and on sick leave. Authors’ conclusions: a high level of work ability was associated with high health-related quality of life, achieved power: the study achieved approximately half of the sample size stated as required for detecting a 10% difference in effect size in the summation of scores of the completed instruments

**Further baseline characteristics of participants** All participants were employed (unemployed participants removed from study sample upon randomization) mean age was 37 (I), 35 (C), slightly fewer females in intervention group 63%(I), 74%f(C), mostly low or middle education 61%(I)**,** 71%(C), antidepressants (based on Eriksson et al. 2017) used by approximately 25% (I) , 21% (C).

**Notes** Means and standard deviations of sickness absence days recalculated for the whole study sample for the purposes of this review, according to instructions recieved in correspondence with the study author Dominique Hange.

**Heber et al. 2016**

**Methods** Same protocol as Ebert et al. 2016. Parallel RCT in Germany, single center, recruitment via occupational health, mai ly through statutory health insurance company BARMER-GEK

**Participants** 264 currently employed adults from general population, with scores 22 on the Perceived Stress Scale (PSS-10)

**Intervention** Eight online multimedia sessions, 45-60min each, containing text, interactive exercises, audio and video files. Performed within seven weeks. Contents based on psycho-education, problem-solving and emotional regulation techniques. Guidance: responses to exercises and reminders by psychologists and trained master’s-level psychology students

**Outcomes** Sickness absence (euros and days) at baseline, post-assessment and at 6 mo. Data was gathered with TiC-P and recall time was the latest 3month period. Sick days are reported for 3months intervals, costs are statistically transformed to a 6month perspective

**Control intervention** Wait-list and access to treatment as usual

**Primary aims and conclusions, achieved power**

Aims: efficacy of internet-based stress management for employees. Authors’ conclusions: Investing in internet-based stress-management interventions is associated with a high probability for a positive financial return. Sample size: achieved power for efficacy of symptom decrease, no calculations for the sickness absence outcome.

**Further baseline characteristics participants** All employed, mean age 42.4 (I), 44.2 (C), mainly females, 73,5 %(I), 72,7% (C), mainly high education: 78.8% (I), 75.0% (C), previous mental health treatment (%I, %C) Previous psychotherapy 39.4%(I), 32.6%(C), current psychotherapy 3.8%(I), 8.3%(C)

**Notes** Some of the authors are stakeholders of the “Institute for Online Health Training" providing the intervention.

**Kraepelien et al. 2018**

**Methods** Parallel RCT in Sweden, with three study arms (iCBT-intervenetion, physical exercise-intervention and control), participants were recruited via multiple primary care clinics, (from a total of six administrative districts).

**Participants** 945 adults with depression in screening ( 10 on the Patient Health Questionnaire-9).

**Intervention** 12 week intervention of self-help text modules, based on CBT principles, 34 modules were available and 30 of these were used selectively (individually tailored treatment), as the participants worked with each module for about a week. The work was guided online by a clinical psychologist or last-year psychology student under supervision. Before treatment, patients also received a short telephone call from this therapist.

**Outcome** Kraepelien et al. 2018 reports sickness absence costs at 12 months. Kaldo et al. 2018 reports categories of sickness absence in days (none, low (1-2), some (3-7) high (8-30), and describes production of estimates from these categories as None= 0 days, Low=1.5 days, Some =5 days, High =19 days (and baseline x12mo, T1 x3mo, T2 x9mo). According to correspondence with Martin Kraepelien, sickness absence data has been collected as self-reported continuous data with a recall time of 4 weeks at baseline and at 12 months, and the categories have been applied to data collection only at the 3-month timepoint.

**Control intervention** Treatment as usual, standard primary care (medication, psychotherapy etc.). 25% of contro group participants recieved no recorded treatments

**Primary aims and conclusions, achieved power** Aims: efficacy and cost-efficacy of intervention. Author conclusions: Kaldo et al. 2018: ICBT in primary care is not more effective in reducing sick leave and unemployment compared with TAU. Achieved Power: Power analyses were designed to find a 10% difference in re-employment or returning after long-term sick leave between any two groups, which with a power of 80% at the 5% level was 330 patients in each arm.

**Further baseline characteristics of participants** 74% were employed, mean age 43.2 (I), 43.2(C), mainly female 72,2%(I), 76,9%(C), high (tertiary) 42% (I), 41%(C) or middle education 54% (I) 54,4%(C), antidepressant use slightly higher in intervention group 30.3(I), 21.7(C) (Kaldo et al. 2018)

**Notes** We attempted to reconstruct the 3 month timepoint dataset using the above mentioned estimates, but had to omit this reconstruction from the final analysis, as it was a source of significant heterogeneity. Instead we hae used the dataset published by Kraepelien et al. 2018 and additional data on the baseline sickness absence communicated by Martin Kraepelien.

**Lindsäter et al. 2019**

**Methods** Crossover RCT in Sweden, single centre study with voluntary recruitment based on advertisements.

**Participants** 100 adults from general population with adjustment disorder (AD) or exhaustion disorder (ED) . (Diagnosis of DSM-5 AD and ICD-10 ED confirmed with a clinical interview).

**Interventions** 12 modules of online self-help text with exercises and homework assignments, performed at a pace of one module per week, the previous module had to be completed to access the next. After 12 weeks the intervention participants continued as in the control intervention. Two licenced clinical psychologists provided feedback on homework assignments and gave emotional and technical support via an asynchronous messaging system. Participants could send questions or concerns to the therapist, and expect an answer within 48 h. Participants also received automated SMS reminders weekly. Outcomes Self-reported (TiC-P) per capita cost in USD (per month?) of sickness absence at baseline and at post-assessment, i.e. during the intervention.

**Control intervention** Control participants were given no treatment during the first 12 weeks of the trial, but filled in the same weekly questionnaires as the intervention group and received automated SMS reminders to do so. After 12 weeks the controls continued as intervention participants (see above).

**Primary aims and conclusions, achieved power** Aims: cost-effectiveness and cost-utility of the intervention. Author Conclusions: ICBT is a cost-effective treatment for patients suffering from AD or ED. Achieved power: sample size was determined a priori for conventional parametric tests to achieve 80% power to detect a between-group difference of d = 0.6, given α = 0.05 (2-tailed).

**Further baseline characteristics of participants** Mostly employed 80% (I), 82% (C), mean age 45.1 (I) 47.2 (C), mainly females 82%(I), 88% (C), mainly high education 88%(I), 94%(C), antidepressant use fairly uncommon, 10%(I) 12%(C),

**Notes** No conflicts of interest

**Persson-Asplund et al. 2018**

**Methods** Parallel RCT in Sweden, recruitment via workplace advertisements directed at an occupational group (managers).

**Participants** 117 first-line and middle managers (adults), with a diagnosis of adjustment disorder (AD) as described in the ICD -10 subdivision F43 Reaction to severe stress, and adjustment disorder.

**Intervention** Eight weekly modules containing text, images, audiofiles and videos, worksheets and other exercises (requiring about 2 3 hours of work/module), based on CBT (behavioral acticvation) and acceptance and commitment therapy. Participants recieved weekly personalised written feedback via email from a psychologist or supervised master-level psychology student on the exercises they had completed.

**Outcomes** Self-reported amount of days absent or present at work while being physically or mentally ill (TiC-P) during the last month (4 week recall time) at baseline, at 8 weeks (after intervention) and at 6 months

**Control intervention** Attention control. Control participants recieved a weekly brief text about a stress-related topic and had a possibility to anonymously discuss each topic with participant peers in a moderated web forum. Controls were also given weekly homework assignments consisting of statements about stress, and received minimal and standardised feedback on their assignments.

**Primary aims and conclusions, achieved power** Aims: Efficacy of the iSMI (internet-based stress management intervention) in distressed managers. Author Conclusions: the iSMI intervention can be an effective, accessible and potentially time-effective approach of reducing stress and other mental-related and work-related health symptoms among distressed managers. Power calculation: Sample size was set to 120 participants. This sample size was estimated with the aim of detecting an effect size (symptom relief) of Cohen’s d=0.41or higher.

**Further baseline characteristics of participants** All employed, mean age 46.9 (I), 46.8 (C), some female majority 72%(I), 63%(C), mostly high education 94%(I), 88% (C), previous stress management interventions or psychotherapy 34% (I), 24%(C).

**Notes** No conflicts of interest declared.

**Romero-Sanchiz et al. 2017**

**Methods** Parallel, three-armed RCT in Spain, comparing a guided and an unguided version of the iCBT intervention to a control group. A multicentre study, with 30 recruiting primary care centres.

**Participants** 296 adults in primary care with a more than two-week history of mild or moderate severity symptoms (scores 14-28) in the Spanish version of the Beck Depression Inventory-II(BDI-II) and no psychological treatment in the last year.

**Intervention** Two initial modules (home and welcome), and eight sequential therapeutic modules based on CBT. Participants were recommended to work on each module for at least one week, with a total duration of the intervention of about three months. A trained psychotherapist contacted patients by email to offer help to overcome difficulties in making progress with the program and technical support.

**Outcomes** Mean total absence cost for one year before baseline and for the 12 mo from randomization in EUR. Sickness absence was self-reported with the Client Service Receipt Inventory, designed to collect retrospective data on service utilization during the previous months after the last assessment.

**Control intervention** Improved treatment as usual. (Participating general practitioners participated in a three-hour training program to update their knowledge of how to treat depression in primary care.)

**Primary aims and conclusions, achieved power** Aims: to examine cost effectiveness and cost utility of the intervention (with and without guidance). Author conclusions: Internet-based CBT interventions are appropriate from both economic and clinical perspectives for depressed patients in the Spanish primary care system. Sample size: Study first aimed at 450 participants, then sample size was recalculated to 300 participants using less conservative criteria.

**Further baseline characteristics of participants** Only 33,1%(I), 34,4%(C) were employed, the mean age was 43.19 (I),43.04 (C), female majority 79.2%f (I) 74.5%f (C), mainly middle-low education 66.7%(I) 70.6% (C), antidepressant medication use at baseline was very common 91.7%(I) 89.2%(C). (Recent psychotherapy among exclusion criteria).

**Notes** No conflicts of interest declared.

**Thiart et al. 2015**

**Methods** Single-center, parallel RCT in Germany, recruitment via advertising invitational emails.

**Participants** 128 primary, secondary or vocational school teachers with insomnia (an Insomnia Severity Index (ISI) score of 15) and work-related rumination ( 15 on the cognitive irritation subscale of the Irritation Scale (IS)), and no ongoing psychological treatment.

**Intervention** Six one-week modules and a 10-item online recovery diary, content based on CBT methods, metacognitive therapy and research on boundary management. Motivational, weekly feedback on exercises was provided via an email messaging system by trained coaches, supervised by a clinical psychologist . Coaches were also available for questions, and provided reminders, with total time spent on each participant not exceeding three hours.

**Outcomes** Absence from work due to sickness was measured at baseline and at 6-months follow-up as the self-recorded total amount of days on sick leave during the past three months.

**Control intervention** Wait-list control

**Primary aims and conclusions, achieved power** Aims: efficacy (and cost-efficacy) of guided internet-based recovery training.Conclusions: Internet based recovery training may help teachers reduce their insomnia symptoms as well as a broad range of related health-outcomes such as rumination and worrying. Achieved power: the sample size of N=128 was sufficient to detect a difference between the study arms at post-test on the primary outcome measure (insomnia severity).

**Further baseline characteristics of participants** All currently employed, mean age 48.4 years (I) 47.6 years (C), mainly females 67.2%f (I) 81.3%f (C)previous psychotherapy 28.1%(I) 43.8%(C)

**Notes** Some authors are stakeholders of the “Institute for Online Health Trainings” founded during the final stages of the study.

***Supplementary Figure 1 iCBT effect on sickness absence in all follow-up timepoints of all included studies***

*Studies in alphabetical order, with all follow-up timepoints presented as separate lines of data in chronological order.*


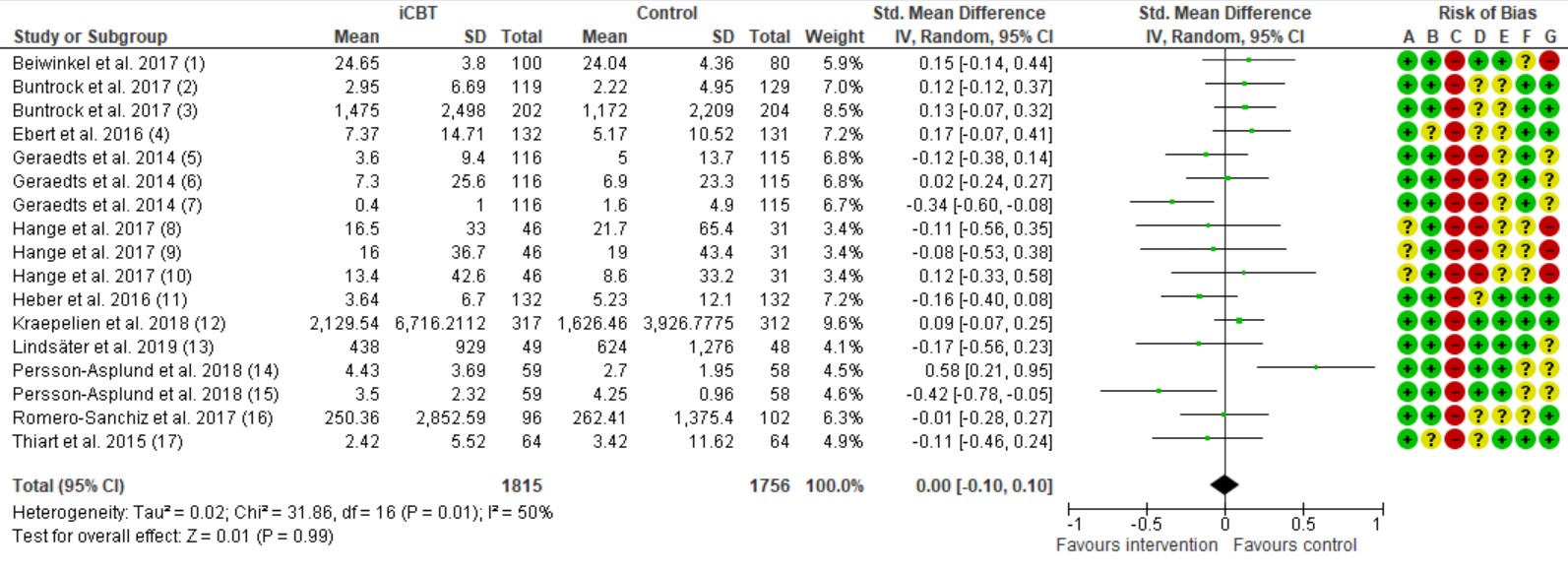


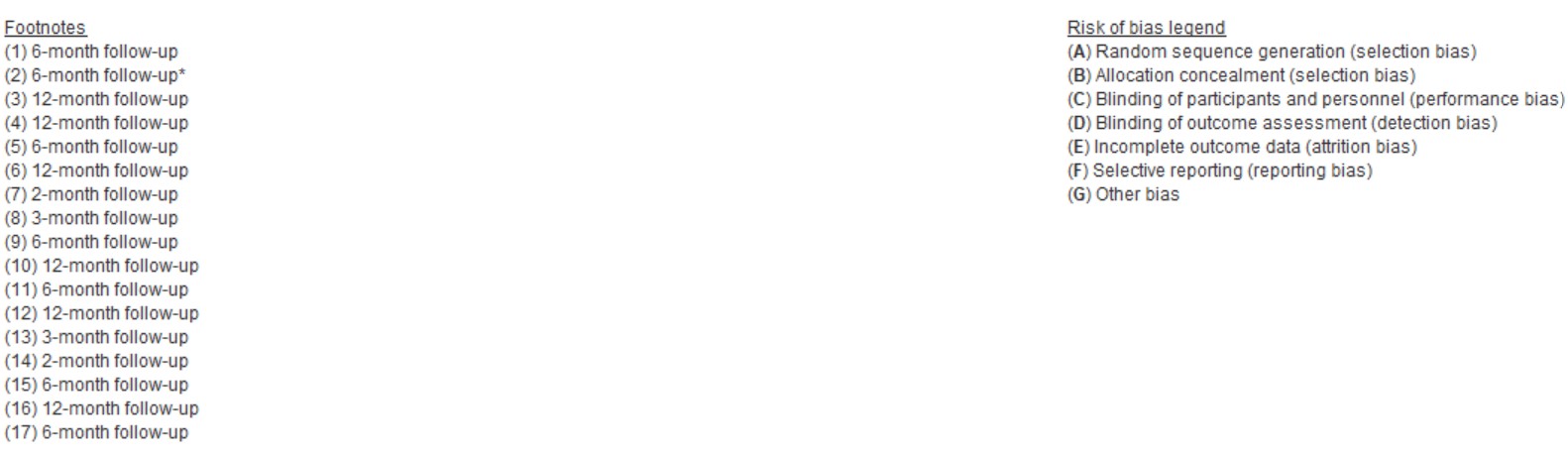


**Supplementary Figure 2** Baseline sickness absence above national average and below the national average


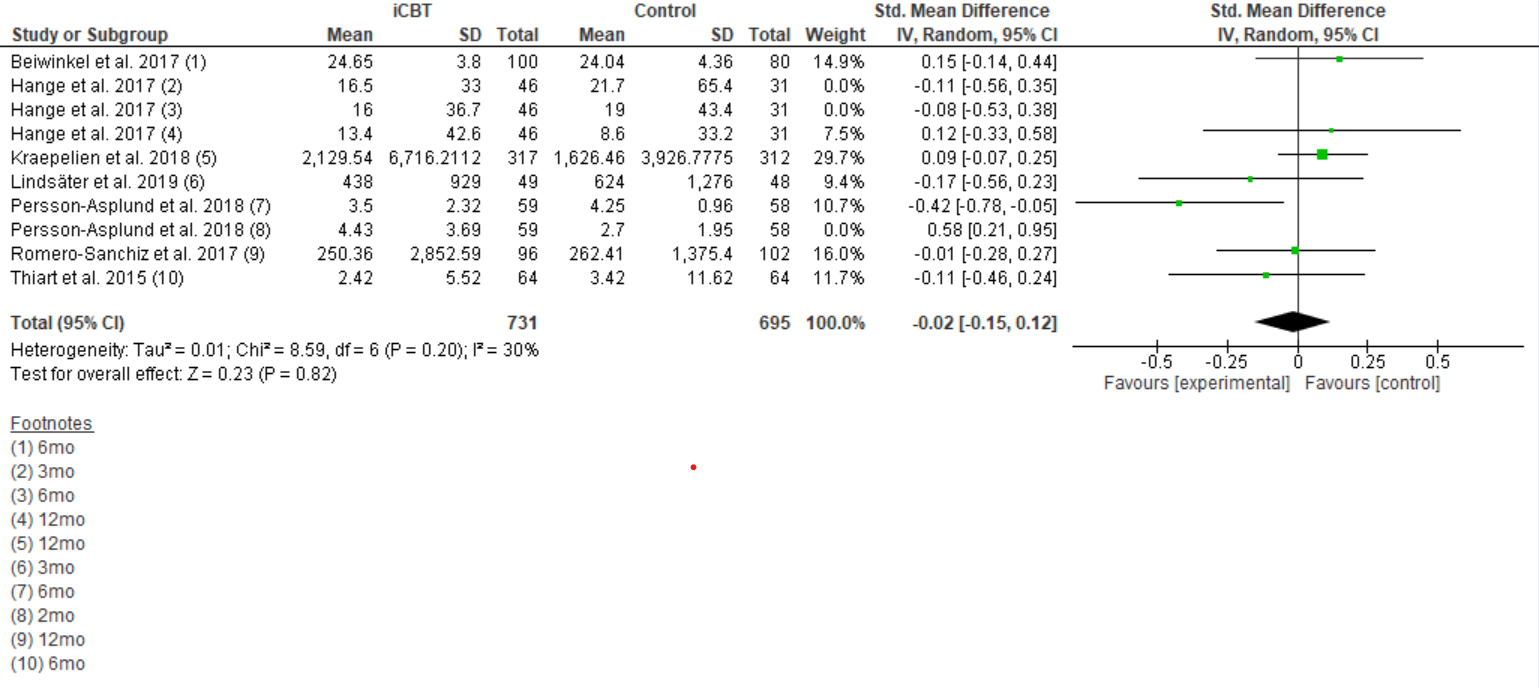


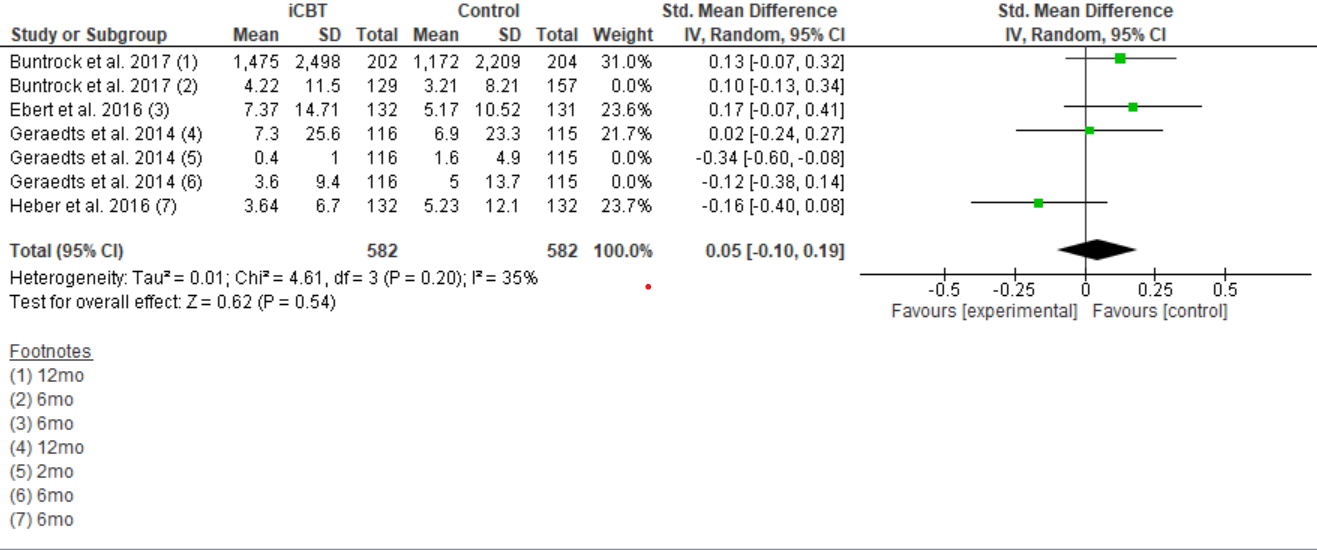

Supplement: sj-docx-4-sjp-10.1177_14034948221075016 – Supplemental material for Internet-delivered cognitive behavioral therapy (iCBT) for common mental disorders and subsequent sickness absence: a systematic review and meta-analysis [file sj-docx-4-sjp-10.1177_14034948221075016.docx]
